# The Role of Astrocyte–Neuron Interactions in Shaping Neuronal Maturation during Human Brain Development

**DOI:** 10.34133/csbj.0083

**Published:** 2026-05-18

**Authors:** Manman Zhao, Ying Zhu

**Affiliations:** ^1^ State Key Laboratory of Brain Function and Disorders, MOE Frontiers Center for Brain Science, Institutes of Brain Science and Department of Neurosurgery, Huashan Hospital, Fudan University, Shanghai 200032, China.; ^2^ Institute of Neuroscience, CAS Center for Excellence in Brain Science and Intelligence Technology, Chinese Academy of Sciences, Shanghai 200031, China.

## Abstract

The human brain undergoes extensive transcriptomic remodeling during the perinatal period and early postnatal development, yet the cellular basis and regulatory logic underlying these changes remain unclear. By integrating single-cell and bulk RNA sequencing data across 9 developmental stages of the human cerebral cortex, we systematically characterized the temporal dynamics of gene expression across major cell types. From late fetal stages to infancy, pronounced transcriptomic changes occur, accompanied by shifts in cellular composition and gene expression programs. During this period, glial cells exhibit gene expression patterns and computationally inferred signaling features consistent with ongoing neuronal maturation. Cell–cell communication analysis further suggests that astrocyte subtypes exhibit distinct interaction patterns with neurons, characterized by subtype-specific temporal dynamics and lineage-associated features. Comparative analyses with mouse brains reveal human-specific features in synaptogenesis and axonogenesis that coincide with the evolutionary divergence of astrocyte subtypes. In addition, immature and mature astrocyte subtypes show distinct associations with neuronal maturation, and their developmental transitions align with stages of increased transcriptomic divergence. These findings provide new insights into how astrocyte subtypes in the postnatal human brain may contribute to neuronal development and circuit formation during critical developmental periods, and offer clues to the potential origins of neurodevelopmental disorders.

## Highlights

What are the main findings?

• Shifts in cellular composition and astrocyte-subtype-specific communication patterns with neurons accompany the increased transcriptomic divergence observed in the human brain from late fetal stages to infancy.
• Astrocytes show transcriptional and inferred interaction patterns associated with neuronal maturation, including subtype-specific interactions with neurons consistent with axonogenesis, synaptogenesis, and neural circuit formation.

What are the implications of the main findings?

• These findings highlight the distinct nature of astrocyte subtypes and their diverse interactions with neurons as features associated with human-specific trajectories of neuronal development and brain circuit formation.
• The temporal transitions of astrocyte subtypes provide a developmental framework for understanding the evolutionary divergence of the human brain and the potential origins of neurodevelopmental disorders.

## Graphical Abstract

**Figure:**
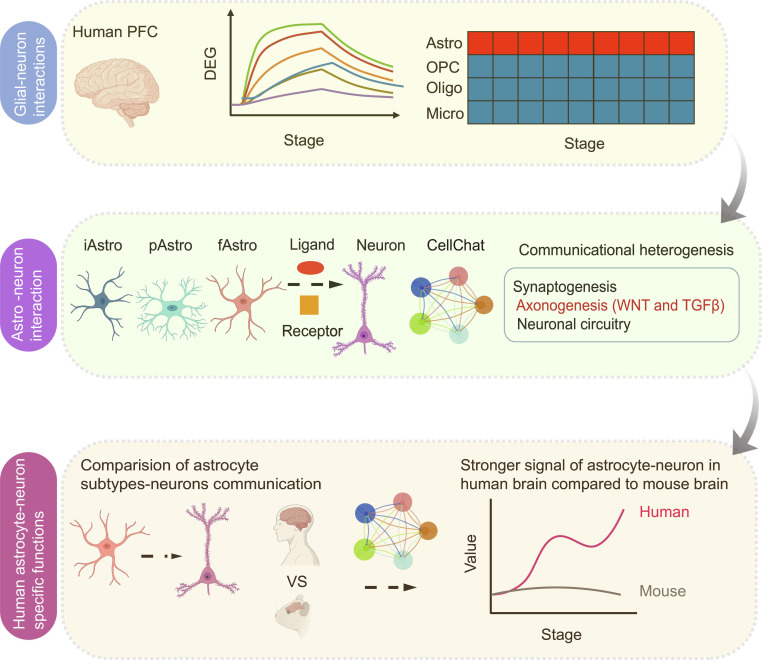

## Introduction

Human brain development is a prolonged and intricate process that encompasses a series of critical neuronal events, including neurogenesis, myelination, synaptogenesis, synaptic maturation, and synaptic pruning [1–3]. While neurons have historically been the focus of developmental studies, glial cells are increasingly recognized as key regulators of neuronal activity. Microglia and astrocytes promote synaptogenesis [4,5], synaptic maturation [6,7], and pruning [8,9], while oligodendrocytes are essential for myelination [10]. However, it remains unclear which glial cell types exert the most prominent regulatory influence on neurons during development, especially in the human brain, where astrocytes not only dominate numerically [11,12] but also display complex morphology [13] and an expanded functional repertoire [14–16].

Astrocytes in the human cerebral cortex comprise multiple morphologically and spatially distinct subtypes, each exhibiting unique transcriptomic profiles [17–19]. The 3 major astrocyte subtypes in the human cortex are, namely, interlaminar (iAstro), protoplasmic (pAstro), and fibrous astrocytes (fAstro). iAstro, a primate-specific subtype, localizes to cortical layers I to II and extends long vertical processes into deeper layers, features that are absent in rodents [20,21]. pAstro, the predominant subtype in gray matter, is conserved across mammals but exhibits more elaborate morphology in humans [22]. fAstro, which predominates in white matter, is structurally simpler and more conserved across species [22]. In addition, the human cortex contains another astrocyte subtype, varicose projection astrocytes (vAstro). vAstro is characterized by long varicose processes and is enriched in deeper cortical layers [23]. Although initially considered unique to humans and chimpanzees, emerging evidence suggests that vAstro may represent a reactive astrocyte subtype associated with neuropathological conditions [22–24]. Despite these morphological and species-specific distinctions, the ways in which astrocyte subtypes interact with neurons during human brain development remain largely unexplored.

In this study, we systematically analyzed the dynamic transcriptomic profiles of glial cells and neurons across human neocortical development to explore glia-specific contributions to neuronal maturation. By integrating single-cell and bulk RNA sequencing (RNA-seq) data, we characterized the timing and nature of transcriptional changes across cell types and examined how shifts in glial transcriptional programs may correspond with functional stages of neuronal development. Using ligand–receptor communication and transcriptional regulon analyses, we computationally inferred that astrocyte–neuron signaling represents the most prominent form of glia–neuron interaction and identified distinct, subtype-specific communication patterns. Collectively, these findings underscore the temporal and subtype-specific roles of astrocytes in shaping human cortical circuits and provide insights into potential species-specific mechanisms underlying brain development.

## Methods

### Data availability and preprocessing

Human brain single-nucleus RNA-seq (snRNA-seq) data of the prefrontal cortex (PFC) were obtained from the public database of the Lister lab [25] and used to examine differentially expressed genes (DEGs), cell–cell communication, and regulon comparisons across lifespan stages. Only genes that were expressed in more than 10 cells were considered. Notably, despite considerable variations in cell numbers across stages, we were able to detect a minimum of 9,000 genes at each stage. Single-cell RNA-seq (scRNA-seq) data of the human cerebral cortex were obtained from the Gene Expression Omnibus (GEO) database (GSE204684 [26]) and used to validate the developmental dynamics in the number of DEGs, as well as to investigate cell–cell communication and regulon comparisons. Bulk RNA-seq data of the human PFC were obtained from PsychENCODE [27] and used to infer cell-type composition across developmental stages, allowing us to examine potential changes in cellular composition during development. Adult human brain snRNA-seq data of the PFC were obtained from the public database [28] and were used to further assess the reliability of astrocyte subtype annotations.

Mouse scRNA-seq datasets of the cerebral cortex during development were obtained from the GEO database (GSE153164 [29] and GSE204759 [30]) and used for comparative analysis with human data on cell–cell communication and regulon comparisons. Additionally, a mouse scRNA-seq dataset of visual cortex development, obtained from the public database of the Zeng lab [31], was used to validate cross-species cell–cell communication and regulon comparisons. Genes detected in more than 10 cells were retained for subsequent analyses. We were able to detect a minimum of 10,000 genes at each stage.

Before performing the cross-species analysis, we conducted orthologous gene mapping and identified 13,447 shared orthologous genes across species.

Proteomics data were sourced from the Kriegstein lab [32]. The raw spatial transcriptome data of the human cerebral cortex generated in this study were obtained from the Genome Sequence Archive under accession code HRA004425.

### Analysis of differentially expressed proteins, DEGs, and cell-type composition

#### DEG analysis across all cell types in human snRNA-seq data

We identified PFC DEGs between adjacent age stages for each cell type using Seurat v5.0.3 [33] (*P* value < 0.05 and |log_2_FC| > 0.5) based on the dataset from the Lister lab [25], with down-sampling applied to ensure robustness. The detailed down-sampling procedure is described in the Supplementary Materials.

#### Correction and evaluation of batch effects in human snRNA-seq data

To further validate the robustness of the observed increase in the number of DEGs from the perinatal to infancy stages, we performed batch-effect correction on the dataset, as the human brain samples were derived from individuals of different ethnicities and sexes. We calculated the average expression level of each gene per cell type in each sample. The gene expression matrix was then corrected for ethnicity and sex using the sva package [34]. Specifically, the full model was defined as model.matrix(~ stages, data = data) to represent the biological variable of astrocyte subtypes, and the null model as model.matrix(~ ethnicities + sexes, data = data) to capture variation associated with ethnicities and sexes. The number of surrogate variables (n.sv) was estimated using the “be” method, and sva was applied to adjust the expression matrix. This approach identifies hidden sources of variation associated with ethnicities + sexes and removes them while retaining stage transcriptomic signals.

Finally, principal component analysis (PCA) was performed for each cell type before and after correction to further assess the patterns of transcriptomic variation.

#### Identification of DEGs between adjacent developmental stages for astrocyte subtypes

For each astrocyte subtype, we identified DEGs between adjacent developmental stages using the rank-sum test method implemented in the Seurat package. Genes were considered markers if they met the thresholds of *P* value < 0.05 and |log_2_FC| > 0.5.

#### Identification of DEPs between adjacent developmental stages

To identify differentially expressed proteins (DEPs) at the tissue level, we utilized publicly available protein expression data [32]. Statistical analysis was performed using the limma package [35], and proteins exhibiting *P* values <0.05 were considered significantly differentially expressed. To ensure robustness, data were preprocessed and normalized according to standard limma workflows prior to differential expression analysis. DEPs were then compared across adjacent developmental stages to characterize dynamic changes in protein expression during human brain development.

#### GO and Kyoto Encyclopedia of Genes and Genomes enrichment analysis

Gene Ontology (GO) enrichment analysis of DEGs was performed using the clusterProfiler package [36], focusing specifically on biological process (BP) terms. This approach allowed us to systematically characterize the key biological processes associated with DEGs identified across developmental stages and cell types. For each DEG set, significantly enriched BP terms were identified based on adjusted *P* values, providing insights into the functional roles of transcriptomic changes observed during brain development. By emphasizing BP enrichment, we aimed to highlight the dynamic biological pathways that underpin cellular and developmental processes in the human brain, thereby supporting the interpretation of downstream analyses, including cell–cell communication.

To investigate the dynamic molecular dialogue between glia and neurons, we utilized the aPEAR package [37] to perform pathway enrichment analysis. This analysis specifically focused on ligand–receptor pairs implicated in their communication across 3 pivotal stages of early brain development: late fetal, neonatal, and infancy. Our goal was to systematically characterize the key signaling pathways and biological processes governing this intricate intercellular cross talk.

#### Cell-type deconvolution

To assess whether changes in cell numbers could influence transcriptomic variations at the bulk level, we performed deconvolution of the bulk RNA-seq data. For each developmental stage, we first created a gene expression matrix and selected the top 30 genes ranked by their principal eigenvalue matrix for each cell type to construct the signature matrix. Cell-type abundances in each sample were then estimated using the CIBERSORT algorithm [38] implemented in R.

### Astrocyte–neuron interaction analysis

#### Analysis of astrocyte–neuron communication

We used CellChat [39] to analyze glia–neuron communication. Communication probabilities were computed based on the average expression of ligands and receptors within each cell group and were evaluated using permutation testing implemented in CellChat, which reduces direct dependence on cell number. To control for potential bias introduced by unequal cell numbers, a stage- and cell-type-specific down-sampling strategy was applied, ensuring that each developmental stage contained the same total number of cells and that each cell type was represented by an equal number of cells across stages.

Signaling pathway activity was inferred using computeCommunProbPathway(), and results were visualized with netVisual_bubble(). We then applied weighted gene coexpression network analysis (WGCNA) [40] to identify modules of signaling pathways with similar activity patterns and performed functional enrichment analysis of the ligand–receptor pairs within each module using the clusterProfiler package.

#### Classification and annotation of astrocyte subtypes

We classified human cortical astrocytes into 3 subtypes: fAstro (high *GFAP* [41], high *AQP1* [28], and high *FOS* [42,43]), iAstro (high *GFAP* [20,41], high *FABP7* [28], and low *FOS* [42,43]), and pAstro (high *SLC1A2* [44,45], high *AQP4*, and low *GFAP* [41]). Fetal iAstro also expressed radial glial markers *CRYAB* and *HOPX* [20]. Subtype identities were validated using spatial transcriptomic data. Specifically, we compared astrocyte markers in spatial transcriptome samples. Among these markers, *GNA14* is primarily expressed in cortical gray matter astrocytes, *SLC39A12* is mainly expressed in gray matter and layer 1 astrocytes, and *SLCO1C1* is expressed in all 3 astrocyte subtypes. Based on the expression of these genes in astrocyte subtypes, we further validated the identities of the astrocyte subtypes. To assess the robustness of astrocyte subtype classification, we employed 2 complementary strategies. First, we performed an integrative analysis with previously published astrocyte subtype datasets [28] using the Seurat package, systematically evaluating the consistency of subtype identities across independent datasets. Second, we assessed cluster stability through 100 iterations of subsampling (80% of cells per iteration) followed by reclustering, calculating Jaccard similarities for each putative astrocyte subtype. Subtypes with a mean Jaccard similarity >0.6 were considered stable and reliably defined.

We classified mouse cortical astrocytes into 3 subtypes: fAstro (high *Gna14*), layer 1 astrocyte (l1Astro) (high *Gfap*, *Aldh1l1*, and *Serpinf1*), and pAstro (high *Gja1*, *Slco1c1*, *Slc39a12*, and *Aqp4*) [46].

To assess differences among astrocyte subtypes, we performed PCA across all developmental stages. We observed that the PC1 was primarily driven by age-related variation rather than subtype-specific differences. To account for this confounding effect, we applied the sva package to remove age-associated batch effects. The full model was defined as model.matrix(~ celltype, data = data) to represent the biological variable of astrocyte subtypes, and the null model as model.matrix(~ stages, data = data) to capture variation associated with developmental stage. The number of surrogate variables (n.sv) was estimated using the “be” method, and sva was applied to adjust the expression matrix. This approach identifies hidden sources of variation associated with age and removes them while retaining subtype-specific transcriptomic signals. To evaluate and separate the sources of variation in astrocyte transcriptomes, we performed the following analyses: (a) Surrogate variable associated genes: for each surrogate variable inferred by sva, we computed the correlation between the surrogate variable and each gene. Genes with a maximum correlation >0.5 and a *P* value <0.05 for any surrogate variable were defined as significantly associated with hidden sources of variation. (b) Age-associated genes: correlations between gene expression and developmental age were calculated, and genes with strong associations (*P* value < 0.05) were considered age related. (c) Subtype-associated genes: to identify genes linked to astrocyte subtypes, we calculated the correlation between each gene and the annotated cell-type labels, selecting genes with significant associations (*P* value < 0.05). (d) Overlap analysis: The 3 sets of genes (surrogate variable associated, age associated, and subtype associated) were then compared to evaluate the extent to which hidden factors or age effects could confound subtype-specific transcriptomic signals. This allowed us to systematically assess the performance of sva in removing unwanted variation while preserving biologically relevant differences.

After removing associated batch effects, we conducted a systematic correlation analysis of transcriptomic data from 3 human astrocyte subtypes and mouse cortical astrocyte subtypes using Pearson’s correlation coefficient. By calculating the correlation coefficients of gene expression profiles between astrocyte subtypes across species, we aimed to compare the transcriptomic differences of astrocyte subtypes between species.

#### Classification and annotation of immature and mature astrocyte subtypes

To distinguish between mature and immature astrocyte subtypes, we utilized specific markers (immature markers: *MEIS2*, *EIF1B*, *NES*, and *FABP7* [47,48]; mature markers: *AQP4*, *S100B*, and *SLC1A2* [48,49]). DEGs were then obtained by comparing immature with mature astrocyte subtypes using Seurat’s rank-sum test with thresholds of *P* value <0.01 and |log_2_FC| >0.5. BP enrichment of DEGs was conducted using the clusterProfiler package.

#### Comparison analysis of astrocyte subtype–neuron interaction in the human brain

To systematically compare communication patterns between astrocyte subtypes and neurons during brain development, we performed the following analyses using CellChat: First, signaling pathways were identified based on comparisons across astrocyte subtypes. Detailed normalization procedures for cross-developmental stage comparisons are provided in the Supplementary Materials. Second, using the WGCNA method, we identified signaling pathway modules with differential activity patterns. Finally, functional enrichment analysis was conducted on the ligand–receptor pairs within each identified module.

#### Cross-species regulon analysis

Using the pySCENIC workflow [50], we identified astrocyte-subtype-specific regulons in human and mouse brains and subsequently performed a cross-species regulon overlap analysis.

Specifically, for the full astrocyte datasets in each species, gene regulatory networks were inferred independently using pySCENIC’s GRN step. This step, based on the transcription factor (TF) list, was used to construct coexpression networks. These networks were then refined using pySCENIC’s CTX step, which employs motif enrichment analysis to prune indirect targets. Finally, regulon activity was quantified at the single-cell level using pySCENIC’s AUCell model. Detailed methods are described in the Supplementary Materials.

Based on regulon activity scores, we applied linear regression models to each astrocyte subtype across developmental stages to identify developmentally associated, subtype-specific regulons. Regulons showing a significant association with astrocyte subtype (*P* value < 0.05, *R*^2^ > 0.2) were retained for further analysis.

A cross-species overlap analysis was then performed to compare subtype-specific regulons between human and mouse astrocytes.

We further conducted BP enrichment analysis of these specific regulons using the clusterProfiler package to systematically investigate the potential biological functions underlying astrocyte subtype differences between species.

#### Cross-species comparison of astrocyte–neuron communication

We analyzed astrocyte–neuron interactions in human and mouse brains, using WGCNA to identify pathway patterns and define functions via ligand–receptor enrichment. To ensure comparability of communication strength across species, we implemented a systematic normalization strategy at both the data input and analysis levels. (a) Data input layer: consistent object construction. For each sample, we used standardized and log-transformed expression values rather than raw counts. The same CellChatDB (a merged version of human and mouse CellChatDB) was applied across all conditions and species. Each dataset was processed independently using identical parameters for subsetData(), identifyOverExpressedGenes(), and related preprocessing steps. (b) Analysis layer: internal normalization for comparability. For each species, communication probabilities and pathway-level networks were computed separately using computeCommunProb(), computeCommunProbPathway(), and aggregateNet(). The individually analyzed CellChat objects were then merged using mergeCellChat() to align cell types and enable direct comparison across conditions and species. Detailed descriptions of the normalization steps across species can be found in the Supplementary Materials.

This dual-layer strategy ensures that communication strengths are calculated consistently, that across-species comparisons are based on aligned cell types, and that potential technical biases arising from differences in sequencing depth or dataset-specific characteristics are minimized. Notably, these comparisons reflect relative trends in communication intensity rather than absolute values, thereby providing a conservative assessment of cross-species differences.

To assess and validate the species-specific variations in synaptic development observed in our analyses, we compiled gene sets related to astrocyte involvement in key neuronal processes, including synaptogenesis, synaptic maturation, and synaptic pruning, based on previously published literature [8,32,51–55]. PCA for these genes was conducted to compare expression between human and mouse brains. These genes are listed in Table S2. For these communication pathway patterns, we further compared human and mouse brains using independent external datasets [26,31].

#### Comparison analysis of immature and mature astrocyte subtypes

Using the WGCNA method, we identified signaling pathway modules with differential activity and performed functional enrichment analysis on the ligand–receptor pairs within each module. By extracting the data from the netVisual_bubble() function, we further analyzed the subtype-specific ligand–receptor pairs involved in communication with neurons, as well as the dynamic changes in the number of all ligand–receptor pairs mediating astrocyte–neuron interactions across subtypes. Additionally, we analyzed DEGs between immature and mature states for each astrocyte subtype using the Wilcoxon rank-sum test implemented in the Seurat package, with filtering thresholds of false discovery rate (FDR) < 0.01 and |log_2_FC| > 0.5. Functional enrichment analysis of the consensus and subtype-specific DEGs for immature and mature astrocytes was then performed using the clusterProfiler package.

#### Validation using external datasets of human brain development

We leveraged the human cortical development dataset from Zhu *et al.* [26], which includes samples spanning infancy, childhood, adolescence, and adulthood, to validate the diverse astrocyte–neuron interactions across neurogenesis, synaptogenesis, neuronal circuit formation, and axon development. This heterogeneity reflects variation across neuronal subtypes, astrocyte subtypes, and astrocyte maturation states. Specifically, we computed eigenpathway values for signaling pathway modules associated with neurogenesis, synaptogenesis, neuronal circuit formation, and axon development and compared their signaling patterns across astrocyte subtypes and maturation states to assess differences in their interactions with neurons.

### Inference of astrocyte subtype trajectories and lineage regulons

Based on uniform manifold approximation and projection analysis results in the Lister lab [25], we observed that astrocytes may follow 3 distinct developmental trajectories. Using the Palantir algorithm [56], a starting point and 3 terminal states were defined to model the developmental progression. This analysis allowed us to assign a pseudotime value to each individual cell, reflecting its relative position along the inferred developmental trajectories.

The RNA velocity along the inferred developmental trajectories was calculated using the scVelo package [57], based on the pseudotime values assigned to each individual cell. This approach allowed us to estimate the direction and speed of transcriptomic changes for each cell along its developmental trajectory.

To validate the inferred trajectories, we applied the PAGA package [58] for astrocyte trajectory inference. Lineage-specific marker genes were identified using the Wilcoxon rank-sum test.

Lineage regulons were identified using pySCENIC. Specifically, for the full astrocyte datasets, gene regulatory networks were inferred independently using pySCENIC’s GRN step to construct coexpression networks. These networks were then refined using pySCENIC’s CTX step through motif enrichment analysis to prune indirect targets. Finally, regulon activity was quantified at the single-cell level using pySCENIC’s AUCell.

Based on regulon activity scores, linear regression models were applied to each lineage across pseudotime to identify developmentally associated and lineage-specific regulons. Regulons showing significant association with lineage subtypes (*P* value < 0.05, *R*^2^ > 0.2) were retained for further analysis.

### Enrichment analysis of psychiatric disorders

Enrichment of psychiatric risk genes in astrocyte–neuron communication modules was assessed using DisGeNET [59]. To evaluate the robustness of our findings, we repeated the enrichment analyses using an independent gene database (GWAS Catalog). For each analysis, Fisher’s exact test was used to assess the enrichment of the set of astrocyte–neuron ligand–receptor pairs within disease-associated genes. To avoid inflation of enrichment significance, the background gene set for Fisher’s exact test was defined as all expressed genes included in the initial cell–cell communication analysis. For each test, a 2 × 2 contingency table was constructed based on the number of overlapping and nonoverlapping genes relative to this total background. Multiple testing correction was performed using the Benjamini–Hochberg procedure, and FDR < 0.05 was considered statistically significant.

## Results

### Temporal transcriptomic dynamics across cell types during human brain development

**Fig. 1.**
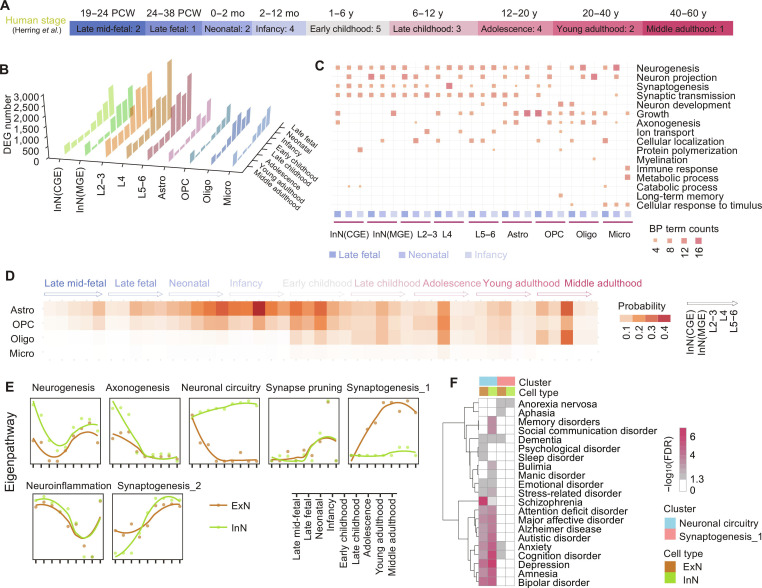
Transcriptomic changes across all cell types and glial–neuronal communication dynamics. (A) Developmental stages with sample sizes. PCW, postconceptional week. (B) Distribution of differentially expressed genes (DEGs) between adjacent developmental stages across cell types at various stages. InN(CGE), inhibitory neurons from caudal ganglionic eminence; InN(MGE), inhibitory neurons from medial ganglionic eminence. L2–3, excitatory neurons in layer 2 and layer 3; L4, excitatory neurons in layer 4; L5–6, excitatory neurons in layer 5 and layer 6; Astro, astrocytes; OPCs, oligodendrocyte precursor cells; Oligo, oligodendrocytes; Micro, microglia. (C) Functional enrichment of DEGs associated with biological processes during pronounced transcriptomic remodeling. Dot size and color denote the number of DEGs enriched in each functional category. All terms met the significance threshold following Benjamini–Hochberg correction (false discovery rate [FDR] < 0.05). (D) Temporal dynamics of glial–neuronal communication across lifespan stages. (E) Astrocyte–neuron signaling pathways were grouped into 7 clusters based on their temporal activity patterns, associated with neurogenesis, axonogenesis, neuronal circuitry, synaptogenesis, synaptic pruning, and neuroinflammation. The *y*-axis indicates the relative activity of each pathway, derived from the principal component analysis of pathway activity. Higher values reflect greater pathway activity relative to other cells or developmental stages. ExN, excitatory neurons; InN, inhibitory neurons. (F) Enrichment of genes related to neuronal circuitry and synaptogenesis in psychiatric and neurological disorders from the DisGeNET database. Enrichment was assessed using Fisher’s exact test, and *P* values were adjusted for multiple testing using the Benjamini–Hochberg method (FDR < 0.05).

Previous studies have reported substantial transcriptomic changes during perinatal and early postnatal human brain development [51,60]. Consistent with these findings, analysis of publicly available protein expression data [32] across the lifespan revealed a similar temporal pattern (Fig. S1A). To further examine whether these changes were primarily driven by alterations in cellular composition or by intrinsic transcriptomic shifts within cell types, we integrated single-cell and bulk RNA-seq data spanning the human PFC across the lifespan [25,27]. Following a previously established framework [51], samples were categorized into 9 distinct developmental stages (Fig. 1A and Table S1). Deconvolution and scRNA-seq analyses revealed pronounced changes in cellular composition during the late fetal, neonatal, and infancy stages (Fig. S1B). In addition, differential expression analysis across adjacent developmental stages identified widespread transcriptomic changes across all major cell types during these periods, consistent with tissue-level trends (Fig. 1B). Down-sampling analyses further indicated that the number of detected DEGs was not biased by cell number or gene number and that up-regulated and down-regulated genes exhibited comparable patterns (Figs. S1C and D S3A). To minimize potential confounding effects, we also performed batch-effect correction for ethnicity and sex while retaining age-related variation. Comparative analyses before and after correction showed that samples were primarily separated along PC1 according to developmental stage (Fig. S2). Notably, samples spanning the perinatal to infancy period displayed pronounced and continuous shifts, suggesting that transcriptomic dynamics are particularly active during this developmental window—consistent with the results of the differential expression analysis. To further validate this observation, we analyzed an independent dataset from the Roussos lab [26], which likewise revealed substantial transcriptomic changes across all major cell types during the same developmental window, thereby further supporting the robustness of our findings (Fig. S1E).

Overall, these analyses suggest that the pronounced transcriptomic change observed at specific developmental stages likely arises from a combination of changes in cellular composition and cell-intrinsic gene expression. Notably, our findings extend prior observations of widespread transcriptomic changes across all cell types during this period, by demonstrating increased transcriptomic divergence within individual cell types during early developmental stages—including the late fetal, neonatal, and infancy periods—although the regulatory mechanisms underlying these changes remain to be further elucidated.

### Glial contributions to neuronal maturation during human brain development

To explore the biological importance of transcriptomic changes during periods of pronounced transcriptomic change, we performed functional enrichment analysis of DEGs across cell types. Distinct functional patterns were observed among glial populations: microglial DEGs were enriched in pathways related to inflammatory responses, whereas oligodendrocyte DEGs were associated with myelination processes. In addition, some glial DEGs were annotated to pathways involved in neuronal development. For example, astrocyte DEGs were enriched in pathways related to synaptogenesis, suggesting a potential supportive role through glia–neuron communication (Fig. 1C and Table S2A). While numerous studies have demonstrated that astrocytes contribute to neuronal synaptogenesis, it is understandable that the majority of this evidence comes from rodents and other model organisms [8,61,62]. Therefore, building on this foundational work, a more thorough examination of these processes specifically in human tissue represents a valuable avenue for further investigation. In this context, our computational analyses offer preliminary evidence that astrocytes may be particularly associated with neuronal synapse formation during periods of pronounced transcriptomic change in the human brain, pointing to a potential stage-specific role that warrants further investigation. However, these annotations may partly reflect shared gene expression programs between glial cells and neurons, rather than direct functional roles in glial cells.

Further analysis of cell–cell communication during these critical stages revealed that ligand–receptor pairs involved in glia–neuron interactions during periods of increased transcriptomic divergence were associated with synapse organization and neuron projection. These results suggest a potential contribution of glial cells to neuronal maturation during stages of pronounced transcriptomic transition (Fig. S4).

### Astrocyte–neuron interactions across the human brain lifespan

We next examined glia–neuron interactions across development, with a particular focus on signaling pathways mediated by ligand–receptor communication. Astrocytes consistently exhibited stronger inferred interactions with neurons than other glial cell types, with these interactions reaching their highest levels during early postnatal development (neonatal and infancy stages) (Fig. 1D). Down-sampling analysis further confirmed that the inferred intercellular communication signal intensity was not biased by differences in cell numbers (Fig. S3B). To more comprehensively characterize glial involvement, we then systematically compared ligand–receptor-mediated communication between neurons and multiple glial populations, including astrocytes, microglia, oligodendrocytes, and oligodendrocyte precursor cells (OPCs). All of these glial cell types showed inferred interactions with neurons in biological processes such as neuroinflammation, axonogenesis, dendritic development, synapse organization, synaptogenesis, and myelination, consistent with previous reports (Fig. S3C). Within this overall pattern, astrocytes displayed relatively stronger inferred signaling associated with dendritic development and synaptogenesis, whereas astrocytes, oligodendrocytes, and OPCs all exhibited comparatively stronger signals related to axonogenesis and myelination.

It is worth noting that the relatively stronger inferred signaling associated with astrocytes may partly reflect their higher cellular abundance and transcriptional activity in the brain. To further explore the potential contribution of astrocytes within the window of increased transcriptomic divergence, we systematically analyzed astrocyte–neuron interactions across developmental stages. Among the identified signaling clusters, one synaptogenesis-related cluster (Synaptogenesis_1) displayed distinct interaction trajectories between astrocytes and excitatory neurons (ExN) compared with inhibitory neurons (InN) (Fig. 1E). Signaling pathways associated with neuronal circuitry remained consistently elevated across development in interactions with InN, whereas in interactions with ExN, these pathways were mainly enriched during the prenatal period and gradually decreased with age. In contrast, the Synaptogenesis_1 cluster, which includes astrocyte–ExN pathways related to synaptogenesis, showed progressively increasing activity across development while remaining consistently low in communication with interneurons at all stages. These distinct interaction patterns were also observed in an independent human brain developmental dataset (Fig. S3D) [26]. Together, these results highlight the cell-type- and stage-specific characteristics of astrocyte–neuron communication and suggest that astrocytes may engage in distinct interaction patterns with excitatory and inhibitory neuronal circuits during brain development.

Notably, both the neuronal circuitry and synaptogenesis clusters were significantly enriched for risk genes associated with a variety of neurological and psychiatric disorders (FDR < 0.05), suggesting potential links between astrocyte-related signaling patterns and disruptions in these pathways. Notably, disease-specific enrichment patterns were observed when comparing astrocyte–interneuron and astrocyte–ExN pathways (Fig. 1F and Fig. S3E). Specifically, disorders such as memory disorders, social communication disorder, bulimia, and manic disorders were primarily associated with astrocyte–interneuron pathways. In contrast, risk genes related to broad psychological disorders and sleep disorders were more enriched in astrocyte–ExN pathways. These findings suggest that astrocyte–neuron interaction patterns may exhibit disorder-specific associations, potentially reflecting differences in the neuronal circuits implicated in each condition.

### Distinct developmental trajectories and communication patterns of astrocyte subtypes

Given the diversity and species-specific features of human astrocytes, we classified them into 3 subtypes—iAstro, pAstro, and fAstro—based on marker gene expression (Fig. S5A to C). To validate the accuracy of this classification, we integrated this astrocyte subtype dataset with previously published astrocyte datasets [28] and observed good concordance between our defined subtypes and those reported in the literature (Fig. S5D and E). We further performed PCA of astrocyte subtypes and, after regressing out age effects, found that the subtypes were well separated along PC1 (Figs. S6 and S7A to C), providing additional support for the robustness of our transcriptome-based definitions. In addition, we assessed the stability of these astrocyte subtypes. While the pAstro subtype demonstrated high clustering stability (mean Jaccard similarity > 0.85), the iAstro and fAstro subtypes exhibited lower stability scores (0.60 to 0.75) (Table S3). This suggests that the transcriptomic profile of pAstro differs substantially from those of iAstro and fAstro, whereas iAstro and fAstro share relatively similar transcriptomic features, which may account for their lower stability scores.

**Fig. 2.**
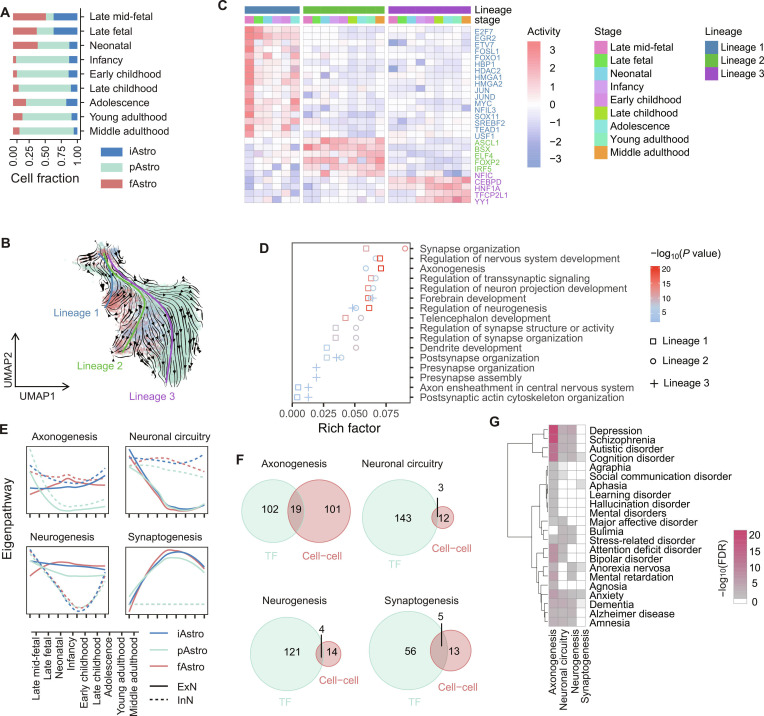
Association of astrocyte subtype developmental lineages with neuronal activity during periods of pronounced transcriptomic remodeling. (A) Depiction of cell fraction alterations in astrocyte subtypes during human brain development. iAstro, interlaminar astrocytes; pAstro, protoplasmic astrocytes; fAstro, fibrous astrocytes. (B) Uniform manifold approximation and projection (UMAP) plot showcasing the developmental trajectory of astrocyte subtypes. The arrows represent the direction of RNA velocity vectors, while the thick blue, green, and purple lines delineate the 3 major cell developmental trajectories. (C) Dynamic transcriptional regulatory activity of transcription factors (TFs) within astrocyte subtype lineage-specific regulons. TFs highlighted in color exhibit lineage-specific regulon TFs. The regulatory activity value of each TF was averaged across cells within each developmental stage, and the resulting values were subsequently *z*-normalized. (D) Functional enrichment of neuronal-activity-related target genes regulated by lineage-specific TFs shown in panel (C). All enriched terms met the significance threshold (false discovery rate [FDR] < 0.05). (E) Astrocyte subtype-to-neuron signal pathways were clustered into 4 different communication patterns, linked to neurogenesis, axonogenesis, neuronal circuitry, and synaptogenesis. The *y*-axis indicates the relative activity of each pathway, derived from the principal component analysis of pathway activity. Higher values reflect greater pathway activity relative to other cells or developmental stages. (F) Overlap between the genes in panel (D) terms and the genes in panel (E) ligand–receptor pairs. (G) Enrichment of genes related to axonogenesis, neuronal circuitry, neurogenesis, and synaptogenesis in panel (E) in psychiatric and neurological disorders from the DisGeNET database. Enrichment was assessed using Fisher’s exact test, and *P* values were adjusted for multiple testing using the Benjamini–Hochberg method (FDR < 0.05).

Developmentally, iAstro and fAstro were primarily generated prenatally, whereas pAstro expanded substantially postnatally (Fig. 2A). Although these 3 subtypes exhibited distinct transcriptomic profiles (Figs. S6 and S7A to C), all showed pronounced transcriptomic change during the late fetal and infancy stages (Fig. S7D). Notably, DEGs during these periods were significantly enriched in pathways related to neuronal development (Fig. S7E), highlighting the potential functional relevance of astrocyte dynamics during early brain development.

RNA-velocity-based trajectory analysis further revealed that pAstro formed a distinct lineage (lineage 3), whereas iAstro and fAstro shared a similar trajectory (lineage 2). In contrast, lineage 1 included all 3 astrocyte subtypes and likely represents proliferative astrocytes (Fig. 2B and Figs. S8A to D and S9). To explore transcriptional programs within these astrocyte lineages, we applied pySCENIC [50] to identify lineage-specific regulons and TFs (see Methods). This analysis uncovered both conserved and lineage-enriched transcriptional programs. Across astrocyte development, a set of conserved TFs, including EGR1 [63], EZH2 [64], HDAC2 [65], and ZEB1 [66], were broadly associated with astrocyte maturation (Fig. S8E). In contrast, there are some TFs that displayed lineage-specific enrichment (Fig. 2C). For example, components of the AP-1 complex, including FOSL1, JUN, and JUND, were enriched in lineage 1 and linked to gene programs associated with cell proliferation and differentiation [67]. Lineage 2, encompassing iAstro and fAstro, was associated with ASCL1, a marker of glial progenitors correlated with astrocyte and oligodendrocyte distribution [68], and FOXP2, notable for its human-specific mutations and expression [69]. Lineage 3, specific to pAstro, showed enrichment of NFIC and YY1, both linked to transcriptional programs involved in cell differentiation [70,71], reflecting distinct trajectory-associated expression patterns.

Genes associated with these subtype-specific transcriptional programs were linked to distinct biological functions. In lineage 2 (iAstro and fAstro), dynamically regulated genes were enriched in pathways related to axonogenesis, synapse organization, neuron projection, and neurogenesis. In lineage 3 (pAstro), regulons predominantly targeted genes involved in synaptogenesis, neuron projection, and neurogenesis (Fig. 2D and Table S2B). Importantly, these enriched pathways primarily relate to neuronal, rather than astrocytic, functions.

To examine potential associations between astrocyte subtypes and neurons, we analyzed predicted communication between astrocyte subtypes and neuronal populations. Clustering of astrocyte subtype–neuron signaling pathways based on developmental interactional activity revealed 8 distinct clusters, primarily linked to axonogenesis, neuronal circuitry, neurogenesis, synaptogenesis, neuronal inflammation, and ion homeostasis, consistent with the overall patterns of astrocyte–neuron signaling (Fig. 2E and Fig. S8F). While most pathways showed similar signaling from different astrocyte subtypes to neurons, with distinct responses between ExN and InN, axonogenesis-related signaling displayed clear subtype-specific heterogeneity. Specifically, iAstro and fAstro exhibited sustained signaling activity throughout cortical development, whereas pAstro showed activity mainly during prenatal and neonatal stages, followed by a decline postnatally. These patterns were consistently observed in independent human brain developmental datasets (Fig. S8G).

We further explored potential associations between astrocyte subtype transcriptomic programs and neuronal signaling by examining overlaps between ligands and receptors in astrocyte–neuron interactions and lineage-specific regulons. This analysis indicated that lineage-associated genes corresponded with inferred ligand–receptor signaling patterns involving astrocyte subtypes and neurons (Fig. 2F). Together, these results reveal subtype-specific astrocyte transcriptional programs and their computationally inferred interactions with neurons, suggesting possible contributions to neuronal development and function.

In the context of astrocyte subtype–neuron communication, genes associated with neuronal circuitry and synaptogenesis showed enrichment for risk genes linked to neurological and psychiatric disorders, consistent with patterns observed in overall astrocyte–neuron communication. Notably, axonogenesis-related genes were enriched across most disorders analyzed, suggesting potential subtype-specific associations with disease susceptibility (Fig. 2G and Fig. S8H).

In summary, astrocyte lineage-associated developmental genes exhibit subtype-specific heterogeneity in axonogenesis and appear to correlate with differential patterns of interaction with ExN and InN. These findings indicate that astrocyte subtypes may have distinct stage- and subtype-associated associations with neuronal programs, without implying a direct regulatory role.

### Human-specific synaptic and axonal functions associated with astrocyte subtype divergence

Subtype-specific developmental lineages of astrocytes in the human brain exhibit distinct molecular signatures and marked heterogeneity in their inferred interaction patterns with neurons during early postnatal development, particularly during axonogenesis (Fig. 2). However, the interspecies divergence of astrocyte subtypes and the corresponding patterns of astrocyte–neuron interactions during these developmental stages remain incompletely understood.

**Fig. 3.**
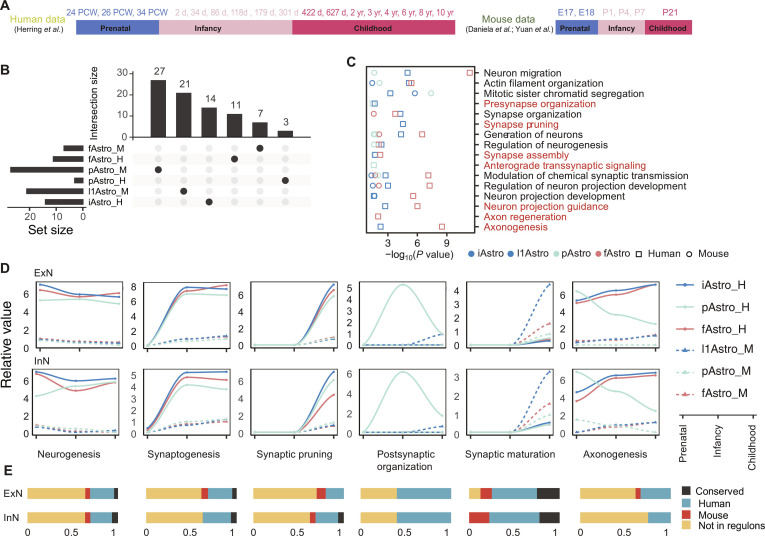
Cross-species comparison of astrocyte subtype divergence. (A) Schematic illustrating the developmental stages and samples of human and mouse. (B) Upset plot depicting shared transcription factors (TFs) in human and mouse astrocyte-subtype-specific regulons, using datasets from the human prefrontal cortex (PFC) and mouse cerebral cortex. iAstro_H, human interlaminar astrocytes; pAstro_H, human protoplasmic astrocytes; fAstro_H, human fibrous astrocytes; l1Astro_M, mouse astrocytes located in layer 1; pAstro_M, mouse protoplasmic astrocytes; fAstro_M, mouse fibrous astrocytes. (C) Functional enrichment analysis of astrocyte-subtype-specific regulons in human and mouse brains. Red labels indicate neuronal functions enriched specifically in humans. All enriched terms met the significance threshold (false discovery rate [FDR] < 0.05). (D) Astrocyte–neuron communication in human and mouse brains across 6 signaling categories. The relative values on the *y*-axis represent the average signaling strength across all pathways involved in communication between astrocyte subtypes and neurons. (E) Overlap analysis between the ligand–receptor pairs within astrocyte subtype–neuron communication modules in panel (D) and human- and mouse-specific regulons of astrocyte subtypes in panel (B). Conserved, ligands and receptors were in both the human and mouse astrocyte-subtype-specific regulons; Human, ligands and receptors were only in the human astrocyte-subtype-specific regulons; Mouse, ligands and receptors were only in the mouse astrocyte-subtype-specific regulons; Not in regulons, ligands and receptors were not in the human and mouse astrocyte-subtype-specific regulons.

To investigate this, we analyzed single-cell transcriptomic datasets from the mouse cerebral cortex (Fig. 3A). Previous studies have classified astrocytes in the mouse cerebral cortex into layer 1 astrocytes and other astrocytic subtypes and have identified their corresponding molecular markers [46]. For instance, *Gfap*, *Aqp4*, *Aldh1l1*, and *Serpinf1* are predominantly expressed in layer 1 astrocytes, while *Gja1* and *Slco1c1* are mainly enriched in astrocytes located in other cortical layers. Building on these findings and incorporating markers of human astrocyte subtypes, we further subdivided mouse astrocytes into 3 categories: layer 1 astrocytes (l1Astro), pAstro, and fAstro (Fig. S10A). These subtypes displayed distinct transcriptomic profiles (Fig. S10B and C). Notably, although l1Astro in mouse were enriched in cortical layer 1, they exhibited a transcriptomic profile distinct from that of human iAstro (Fig. S10D).

To further investigate potential regulatory differences across species, we constructed subtype-specific regulatory networks for human and mouse astrocytes using pySCENIC. This analysis suggested apparent interspecies divergence, with minimal overlap in identified TFs between species (Fig. 3B and Fig. S10F). However, we emphasize that such differences may be strongly influenced by methodological factors, including ortholog mapping strategies, regulon inference parameters, and variability in dataset quality and coverage. As such, these results should be interpreted cautiously and not taken as direct evidence of evolutionary divergence.

Functional enrichment analysis indicated that regulons in both human iAstro and fAstro were associated with pathways related to synapse assembly, neuron projection guidance, axonogenesis, and synaptogenesis (Fig. 3C and Table S2C). Additional enrichments were observed in specific human astrocyte subtypes, such as synapse pruning in iAstro and axon regeneration in fAstro, as well as presynapse organization in pAstro and iAstro. In contrast, mouse astrocyte regulons did not exhibit similarly distinct enrichment patterns, although many functional categories overlapped with those identified in human. No clearly mouse-specific enriched pathways were identified. Importantly, these differences may reflect limitations in analytical sensitivity, annotation completeness, or cross-species comparability, rather than true biological differences.

Astrocyte–neuron communication was next examined by analyzing interaction strength patterns across developmental stages and species. This analysis identified 6 functional clusters, including neurogenesis, axonogenesis, and 3 clusters associated with synapse development (Fig. 3D and Fig. S10E). While astrocyte–neuron communication appeared generally higher in the human brain across several clusters, synaptic maturation showed relatively stronger interaction signals in the mouse brain. To further assess these patterns, we curated 3 gene sets from the literature related to synaptogenesis [8,51–55], synapse maturation [32,51,52], and synaptic pruning [8,51,52] and examined their expression dynamics across developmental stages. These patterns were broadly consistent with the cell–cell communication analysis (Fig. S10G and Table S4) and were supported by additional comparative datasets (Fig. S10H) [26,31]. Nevertheless, these analyses remain correlative and are considered hypothesis generating.

Notably, postsynaptic organization appeared more prominently associated with human pAstro, and human pAstro showed distinct predicted communication patterns with both ExN and InN during axonogenesis. In contrast, mouse astrocyte subtypes exhibited more limited cross-subtype variation, primarily in interactions with InN. Analysis of ligand–receptor pairs suggested that inferred regulon differences may contribute to variation in receptor and ligand expression (Fig. 3E). However, given the reliance on computational inference and cross-species integration, it remains unclear whether these observations reflect genuine biological divergence or methodological artifacts. Further experimental validation will be required to clarify these possibilities.

### Distinct interaction patterns of immature and mature astrocytes during neuronal maturation

As noted above, astrocyte-to-neuron signaling exhibited clear temporal specificity. To further investigate this phenomenon, we examined when astrocyte subtypes transition from immature to mature states during development and whether communication dynamics with neurons differ between these 2 states.

**Fig. 4.**
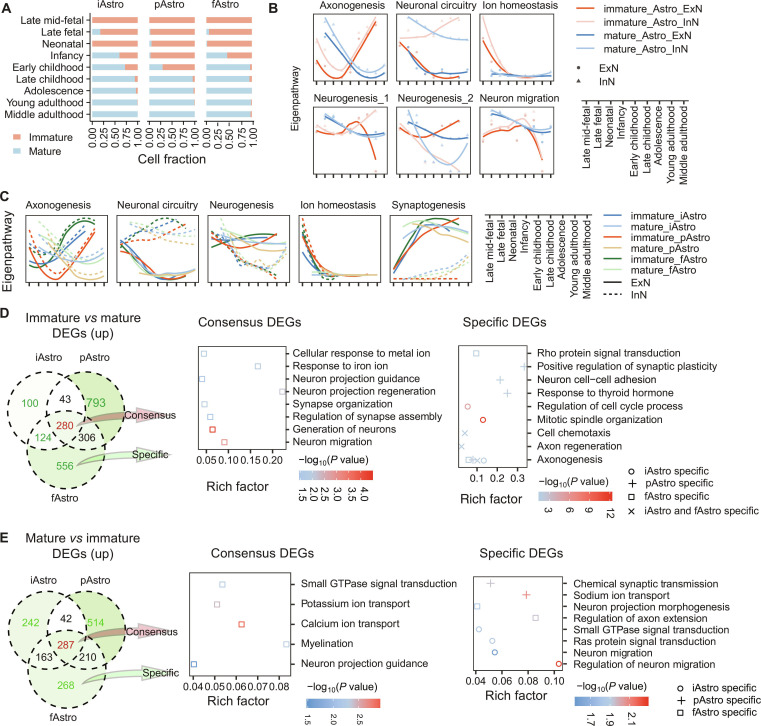
Comparative analysis of neuronal communication between immature and mature astrocytes. (A) Distribution of cell numbers for immature and mature astrocyte subtypes across developmental stages. (B) Communication between immature and mature astrocytes and neurons across 6 pathway types, associated with axonogenesis, neuronal circuitry, ion homeostasis, neurogenesis, and migration. The *y*-axis indicates the relative activity of each pathway, derived from the principal component analysis of pathway activity. Higher values reflect greater pathway activity relative to other cells or developmental stages. (C) Communication between immature and mature astrocyte subtypes and neurons across 5 pathway types, associated with axonogenesis, neuronal circuitry, neurogenesis, synaptogenesis and ion homeostasis. The *y*-axis indicates the relative activity of each pathway, derived from principal component analysis of pathway activity. Higher values reflect greater pathway activity relative to other cells or developmental stages. (D and E) Overlap of highly expressed markers between immature and mature astrocyte subtypes, together with functional enrichment of consensus genes (numbers in red) and subtype-specific genes (numbers in green). All enriched terms reached the significance threshold (false discovery rate [FDR] < 0.05).

Immature and mature astrocyte subtypes were distinguished based on the expression of the immature markers *MEIS2*, *EIF1B*, *NES*, and *FABP7* and the mature markers *AQP4*, *S100B*, and *SLC1A2* (Fig. S10I). The maturation of astrocyte subtypes showed distinct temporal patterns during human brain development. Specifically, the iAstro and fAstro subtypes began transitioning from immature to mature states primarily during infancy, whereas maturation of the pAstro subtype occurred later, beginning in early childhood (Fig. 4A).

Concomitant with astrocyte maturation, inferred astrocyte–neuron interactions exhibited substantial temporal changes. Signals predicted from both immature and mature astrocytes were associated with processes including axonogenesis, neuronal circuitry, ion homeostasis, neurogenesis, and neuronal migration (Fig. 4B). Among these processes, axonogenesis-related signaling displayed a pronounced V-shaped temporal pattern. Specifically, signals from immature astrocytes reached their lowest level during the neonatal period, while signals from mature astrocytes declined again during adolescence. These dynamics suggest that immature astrocytes may be more closely associated with early stages of axonal development, whereas mature astrocytes may participate in axonogenesis-related interactions during later stages of circuit refinement (Fig. 4B and Fig. S10J). Additionally, astrocyte-to-neuron signals associated with neuronal circuitry were predicted to be stronger toward InN than toward ExN, indicating a potential cell-type preference in these inferred interactions.

Subtype-level analysis further showed that the V-shaped pattern of axonogenesis-related signaling was primarily driven by pAstro. Both immature and mature pAstro exhibited biphasic trajectories, with distinct minima occurring at the neonatal and adolescent stages, respectively (Fig. 4C). In contrast, iAstro and fAstro displayed a crossover pattern: immature cells showed relatively higher axonogenesis-related signaling during early development, whereas mature cells exhibited lower levels after infancy. Together, these findings suggest that astrocyte subtypes are associated with temporally distinct axonogenesis-related interaction patterns, potentially reflecting differences in their developmental origins and maturation timelines. Other signaling pathways, including those related to synaptogenesis and neuronal circuitry, appeared less dependent on astrocyte subtype or maturation state but still showed notable cell-type-associated patterns, particularly in the differential inferred communication with InN versus ExN.

To further explore transcriptomic changes associated with astrocyte maturation, we identified genes specifically enriched in immature or mature astrocytes across subtypes. A total of 280 genes were identified as immature astrocyte markers shared across subtypes (Fig. 4D and Table S2D). These genes were significantly enriched in processes such as synapse organization, synaptogenesis, neurogenesis, neuron migration, and neuron projection development, indicating a broad association of immature astrocytes with early stages of neuronal wiring and maturation. Interestingly, subtype-specific genes of all immature astrocyte subtypes were also enriched in axonogenesis, consistent with the observed divergence in predicted astrocyte–neuron communication. In contrast, mature markers shared across subtypes (*n* = 287 genes) or subtype-specific markers were enriched in pathways including small GTPase signal transduction and ion transport, reflecting a shift in the inferred roles of astrocytes toward interactions with more mature neural circuits (Fig. 4E and Table S2D).

**Fig. 5.**
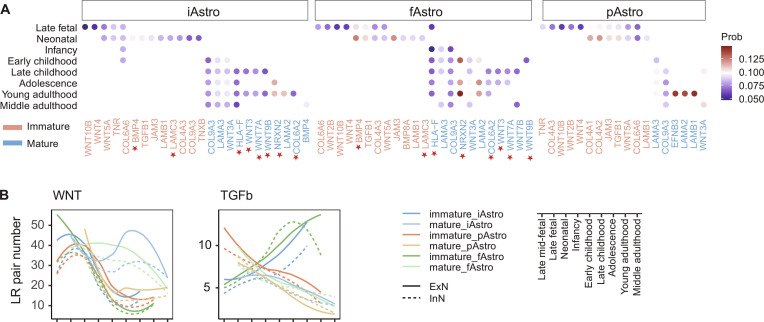
Ligand–receptor pairs and signaling pathways associated with axonogenesis. (A) Ligand signaling enriched in immature and mature astrocyte subtypes that interact with other receptors when comparing the 2 cell states. Prob, interaction probability. Red stars denote ligand signals shared between iAstro and fAstro but distinct from those in pAstro. (B) Dynamic changes in the number of ligand–receptor pairs within the WNT and TGFβ signaling pathways across all astrocyte subtypes associated with axonogenesis during development.

To investigate the molecular programs associated with subtype-specific axonogenesis-related interactions, we analyzed axonogenesis-related ligand–receptor pairs enriched in predicted interactions between immature and mature astrocyte subtypes and neurons. Overall, signals enriched in immature and mature astrocytes in iAstro resembled that in fAstro but differed more substantially from those in pAstro (Fig. 5A). While some pathways, such as WNT, transforming growth factor beta (TGFβ), and collagens, exhibited immature or mature enrichment patterns across all subtypes, distinct WNT ligands were involved in pAstro compared to iAstro/fAstro. Specifically, mature pAstro expressed some WNT ligands (e.g., WNT3A), whereas immature pAstro predominantly expressed other WNT ligands (WNT4 and WNT2B) (Fig. 5A and Fig. S11). In contrast, immature iAstro and fAstro were enriched for ligands like WNT4 and WNT10B, while their mature counterparts were enriched for WNT9B, WNT7A, and WNT3. Furthermore, iAstro and fAstro shared signaling pathways distinct from pAstro, including BMP4 and LAMC3 in the immature stage and HLA-F, WNT3, WNT7, WNT9, NRXN2, and COL6A2 in the mature stage. The subtype-specific axonogenesis-related signaling appeared to involve shared pathways that were associated with distinct ligand–receptor pairs in mature and immature astrocytes of different subtypes, or common signaling pathways showing divergent patterns across subtypes. For instance, WNT signaling exhibited differing communication strength patterns across developmental stages in mature iAstro/fAstro compared to mature pAstro (Fig. 5B). Conversely, TGFβ signaling, although using the same ligand–receptor pairs across subtypes, exhibited unique temporal patterns in pAstro compared to those in iAstro and fAstro, mirroring the developmental trajectory of astrocyte subtype-to-neuron signaling intensity during axonogenesis.

Together, these findings highlight that astrocyte maturation is not solely a cell-intrinsic process but is associated with temporally patterned, subtype-specific signaling programs that correlate with the development of neuronal circuits.

## Discussion

This study shows that the human brain transcriptome undergoes increased transcriptomic divergence from the perinatal period through infancy. Although this phenomenon has been reported previously [25,60], the potential mechanisms underlying these changes have not been systematically investigated in the human brain. The present analysis suggests that these pronounced transcriptomic shifts largely reflect dynamic changes in cellular composition together with cumulative transcriptional alterations within individual cell types. Potential confounding influences arising from dataset heterogeneity and technical variability were carefully considered and controlled in our analyses. Previous studies have linked transcriptional changes during this developmental window to neurodevelopmental processes such as axonogenesis, axon guidance, and synaptic signaling [60]. Extending these observations, we found that astrocytes were uniquely specialized in signaling for dendritic development and synaptogenesis; moreover, a broader coalition of astrocytes, oligodendrocytes, and OPCs was engaged in signaling for axonogenesis and myelination.

In the GO enrichment analysis of glial DEGs, several enriched terms were related to neuronal functions. However, because astrocytes and neurons share the expression of many genes [72–75], this enrichment may partly reflect shared transcriptional programs rather than glial–neuronal communication functions. Thus, these results are interpreted here as indicative of potential functional associations rather than direct evidence of glial activity.

We further systematically investigated astrocyte–neuron communication and its developmental dynamics in the human brain. The analyses suggest that astrocytes exhibit stronger predicted neuronal interaction signals compared with other glial cell types. However, several limitations should be considered when interpreting these results. First, the CellChat framework is sensitive to gene expression quantification, meaning that sequencing quality can influence the inferred communication networks. Second, the number of samples across developmental stages is uneven, which may confound biological differences with technical variation such as sequencing depth. Third, astrocyte abundance peaks during the perinatal and early postnatal periods, and therefore, increased communication signals detected at these stages may partially reflect changes in cell abundance rather than solely subtype-specific properties. Finally, ligand–receptor databases remain incomplete and may not capture all biologically relevant interactions. Consequently, the communication patterns described here should be interpreted as potential interaction landscapes rather than direct evidence of *in vivo* signaling activity. Future studies incorporating experimental validation, including perturbation of candidate ligand–receptor interactions and functional assays in cellular or animal models, will be required to determine whether these predicted interactions correspond to biologically relevant signaling mechanisms.

For astrocyte subtype classification, we defined 3 subtypes—iAstro, fAstro, and pAstro—based on characteristic marker gene expression profiles. Through stability assessment of astrocyte subtype definitions, we observed that the pAstro subtype exhibited a mean Jaccard similarity above 0.85, suggesting a relatively stable cluster. In contrast, the iAstro and fAstro subtypes showed mean Jaccard similarities between 0.60 and 0.75, which fall into what has been described as the “doubtful” category in previous literature. This may indicate that while some patterns exist within these subtypes, the assignment of individual cells remains less certain. It is therefore possible that these 2 subtypes share similar transcriptomic profiles, as they may correspond to astrocyte subtypes that have been previously identified in adult primates and rodents [20]. We acknowledge that further experimental validation would be helpful to clarify their nature. We did not identify a vAstro population based on canonical marker genes. Several factors may explain this observation. First, sequencing depth may have been insufficient to robustly detect markers specific to this population. Second, the overall number of profiled cells may have limited our ability to capture rare cell types. In addition, previous studies suggest that vAstro cells may exhibit considerable inter-individual heterogeneity and may preferentially arise under inflammatory or stress-related conditions while being largely absent under normal physiological states [23,24,76,77]. Notably, both the definition and even the existence of this proposed population remain controversial. Future studies with deeper sequencing and complementary experimental approaches will be required to determine more conclusively whether vAstro cells represent a distinct astrocyte population.

These 3 subtypes (iAstro, fAstro, and pAstro) further exhibited distinct developmental trajectories and predicted communication patterns. While iAstro and fAstro exhibited broadly similar signaling profiles, both differed from pAstro, particularly in pathways associated with axonogenesis and neurogenesis. These distinctions may reflect differences in developmental origin. Consistent with this possibility, RNA velocity and lineage trajectory analyses suggest that astrocyte subtypes likely represent transcriptional states along developmental, spatial, and maturation continua rather than discrete, functionally validated cell populations. Further analysis of ligand–receptor interactions revealed different regulatory patterns among astrocyte subtypes. Variations in axonogenesis-related signaling from iAstro and fAstro were primarily associated with changes in the number of ligand–receptor pairs, whereas variation in pAstro appeared to be more closely related to differences in the specific ligand–receptor pairs involved. In addition, the analyses suggest that iAstro and fAstro may be associated with neuronal maturation through WNT and TGFβ signaling pathways. Previous studies have demonstrated that WNT signaling molecules (e.g., WNT7A [78,79]) and members of the TGFβ family (e.g., TGFB1 [80,81]) contribute to the regulation of neuronal maturation, consistent with our observations. However, these findings are currently based on computational inference, and it remains unclear whether iAstro and fAstro directly influence neuronal maturation through secretion of WNT7 or TGFB1. Experimental validation using *in vitro* and *in vivo* systems will therefore be necessary to test this hypothesis in the future. If confirmed, such findings would provide important insights into the functional contributions of astrocyte subtypes to neuronal maturation.

Within the astrocyte population, we also identified 3 major developmental lineages. One lineage, characterized by strong proliferative capacity and expression of neural stem cell markers, terminated during childhood. This lineage likely represents astrocytes derived directly from ventricular radial glia [82], which are known to generate astrocytes with stemlike properties. The transient presence of this lineage suggests a temporally restricted role during early neurodevelopment, although whether these stemlike astrocytes persist into later developmental stages and influence neuronal activity remains to be determined.

Cross-species comparative analysis revealed substantial divergence in the developmental programs of astrocyte subtypes between humans and mice. These species-specific features may contribute to differences in neural development through several mechanisms, including distinct transcriptional regulatory networks, divergent spatiotemporal patterns of synaptic maturation, and differences in circuit architecture. Notably, human-specific astrocyte regulons were enriched for genes associated with axonogenesis and synaptogenesis, highlighting potential unique aspects of human cortical development. These transcriptomic differences should be viewed as indicative rather than definitive, since they could arise from true biological variation or from technical factors related to regulon inference. Distinguishing between these possibilities will be an important step for future experimental validation. Furthermore, cross-species comparisons of astrocyte–neuron communication strength varied depending on the dataset analyzed. Human data from the Lister lab showed relatively stronger communication signals compared with mouse data, whereas human data from the Zhu dataset displayed only modest differences. Such variability likely reflects differences in sequencing depth and other technical factors across datasets.

Abnormal communication between astrocytes and neurons has increasingly emerged as a central focus in the study of neuropsychiatric disorders. Traditionally, astrocytes were regarded primarily as supportive cells; however, accumulating evidence indicates that they actively influence neural circuit function through mechanisms such as gliotransmitter release, regulation of ionic homeostasis, and modulation of metabolic support [83–85]. Several astrocyte–neuron signaling pathways were identified in which ligand–receptor interactions associated with InN-related circuits were enriched in genes linked to a broad range of neuropsychiatric disorders. Notably, these interactions did not show clear preferential associations with specific categories of disorders, such as neurodevelopmental disorders, affective disorders, or primary psychotic disorders. Based on these observations, we speculate that astrocytes may influence neuropsychiatric disease-related processes preferentially through interactions with InN rather than ExN. In addition, when examining communication between astrocyte subtypes and neurons, we observed subtype-specific differences in signaling pathways associated with axonogenesis. During postnatal stages, these interactions were predominantly associated with iAstro and fAstro, and disruptions in these signaling interactions may potentially be related to the development of disorders such as autism spectrum disorder, depression, and schizophrenia. By contrast, pAstro–neuron communication may primarily contribute to physiological functions that support normal brain function and survival, whereas signaling interactions involving iAstro and fAstro may be more closely associated with processes that, when dysregulated, could contribute to neuropsychiatric disease vulnerability.

It is worth considering several factors when interpreting the results of the CellChat analysis. First, communication scores are calculated as the product of averaged ligand and receptor expression levels. This approach implicitly assumes that expression abundance reflects signaling strength—an assumption that may not always hold, given potential influences such as posttranscriptional regulation, protein degradation, or receptor internalization. Second, while we have normalized data across developmental stages and species using centered log-ratio transformation, we acknowledge that this method may not fully account for batch effects related to sequencing depth or differences in cell-type composition. Third, the ligand–receptor pairs used for inference are primarily derived from existing databases (e.g., CellChatDB), which may offer less comprehensive coverage for nonhuman species or less characterized developmental stages. Fourth, as with most single-cell data, the lack of spatial resolution means that inferred interactions might involve cell types that are not physically adjacent in the tissue. Accordingly, we view these predictions as a prioritized list of candidate interactions that would benefit from experimental validation.

Overall, this study provides a systematic characterization of transcriptionally and potentially functionally distinct astrocyte subtypes, revealing subtype-associated developmental programs, lineage relationships, cross-species divergence, and stage-dependent astrocyte–neuron interactions during brain development. Nevertheless, several limitations should be acknowledged. First, astrocyte subtypes were identified solely through computational annotation of scRNA-seq and snRNA-seq datasets without experimental isolation. These subtypes therefore likely represent transcriptional states distributed along developmental, spatial, or maturation continua rather than fully discrete cell populations. Second, the limited availability of human brain samples, together with the technical challenges associated with isolating specific astrocyte subtypes, currently restricts opportunities for direct experimental validation. Third, the disease enrichment analyses presented here suggest potential associations but remain inherently inferential. Such analyses may be influenced by factors including heterogeneity of disease gene lists, background gene selection, and multiple testing correction. Consequently, direct mechanistic links between astrocyte subtypes and disease etiology cannot be established at this stage and will require further experimental investigation.

The primary contribution of our study lies in the characterization of the temporal dynamics of transcriptomic remodeling and subtype-specific astrocyte–neuron interactions. While astrocyte functions in neuronal development have been extensively documented, our analysis aims to further refine this framework by examining these dynamic and cell-type-specific features in the human cortex. Comparative analyses also suggest that some of these patterns may be differentially represented across species, potentially relating to astrocyte diversification during evolution. We note, however, that our conclusions are primarily based on computational inference from transcriptomic data and therefore may be influenced by dataset-specific and methodological limitations. Experimental validation will be necessary to further assess the functional relevance of the proposed interactions and mechanisms. Taken together, these findings extend current understanding while remaining consistent with established biological knowledge.
