# Supplementary material for: The Role of Astrocyte–Neuron Interactions in Shaping Neuronal Maturation during Human Brain Development
Source: Comput Struct Biotechnol J. 2026 May 18;2026issue-1(1):0083. doi: 10.34133/csbj.0083 (PMC13181173; doi:10.34133/csbj.0083)
Supplement: Supplementary 1 — Supplementary Text Tables S1 to S5 Figs. S1 to S11 [file csbj.0083.f1.zip › FigS4.pdf]

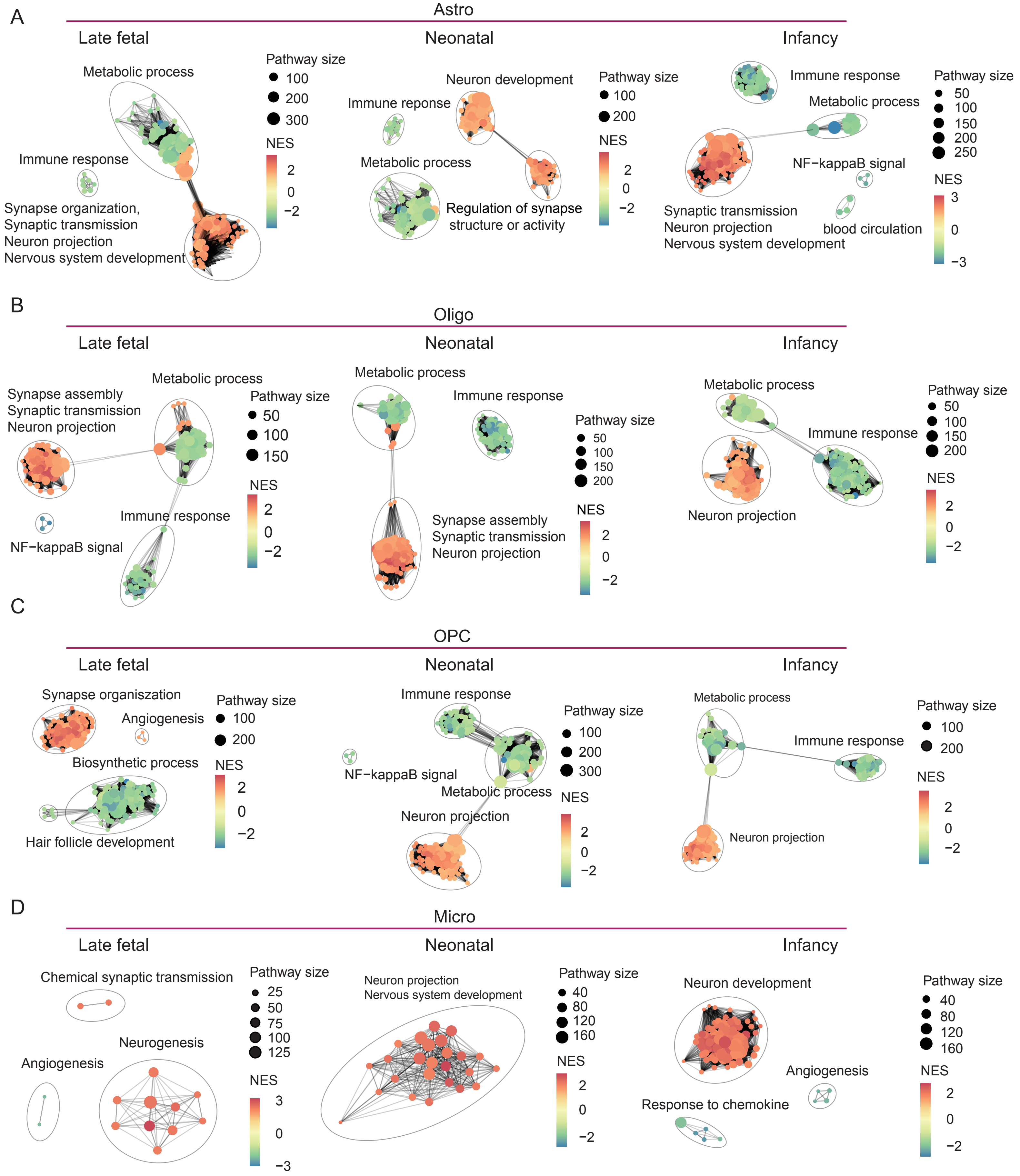

A
Astro
Late fetal
Neonatal
Infancy
Metabolic process
Pathway size
100
200
300
NES
2
0
−2
Immune response
Synapse organization,
Synaptic transmission
Neuron projection
Nervous system development
Neuron development
Immune reponse
Pathway size
100
200
NES
2
0
−2
Metabolic process
Regulation of synapse
structure or activity
Immune response
Pathway size
50
100
150
200
250
Metabolic process
NF−kappaB signal
NES
3
0
−3
Synaptic transmission
Neuron projection
Nervous system development
blood circulation
B
Oligo
Late fetal
Neonatal
Infancy
Metabolic process
Synapse assembly
Synaptic transmission
Neuron projection
Pathway size
50
100
150
NES
2
0
−2
Immune response
NF−kappaB signal
Metabolic process
Immune response
Pathway size
50
100
150
200
NES
2
0
−2
Synapse assembly
Synaptic transmission
Neuron projection
Metabolic process
Immune response
Pathway size
50
100
150
200
NES
2
0
−2
Neuron projection
C
OPC
Late fetal
Neonatal
Infancy
Synapse organiszation
Angiogenesis
Pathway size
100
200
Biosynthetic process
NES
2
0
−2
Hair follicle development
Immune response
Pathway size
100
200
300
NF−kappaB signal
Metabolic process
NES
Neuron projection
2
0
−2
Metabolic process
Pathway size
100
200
Immune response
NES
2
0
−2
Neuron projection
D
Micro
Late fetal
Neonatal
Infancy
Chemical synaptic transmission
Pathway size
25
50
75
100
125
Neurogenesis
Angiogenesis
NES
3
0
−3
Neuron projection
Nervous system development
Pathway size
40
80
120
160
NES
2
0
−2
Neuron development
Pathway size
40
80
120
160
Angiogenesis
NES
Response to chemokine
2
0
−2
