# Supplementary material for: The Role of Astrocyte–Neuron Interactions in Shaping Neuronal Maturation during Human Brain Development
Source: Comput Struct Biotechnol J. 2026 May 18;2026issue-1(1):0083. doi: 10.34133/csbj.0083 (PMC13181173; doi:10.34133/csbj.0083)
Supplement: Supplementary 1 — Supplementary Text Tables S1 to S5 Figs. S1 to S11 [file csbj.0083.f1.zip › Supplementary Materials.docx]

# Supplementary Material

## Down-sampling for DEGs analysis

To address potential biases arising from differences in cell population sizes across developmental stages, we employed a down-sampling approach to evaluate the robustness of the identified DEGs. Briefly, for each cell type, we first assessed the number of cells at each age stage and identified the stage with the smallest cell count, which then served as the reference for down-sampling. For each pair of adjacent developmental stages, cells were randomly subsampled to match this minimum count, and DEGs were detected using the rank sum test. To ensure reliability and account for sampling variability, this procedure was repeated ten times, and genes consistently identified across iterations were considered robust DEGs. This strategy allowed us to minimize potential confounding effects caused by unequal cell numbers while providing a conservative estimate of stage-specific transcriptional changes.

## Communicational normalization

To account for differences in sequencing depth and cell number across samples, CellChat internally normalizes gene expression using a centered log-ratio (CLR) transformation per cell group. However, to further control for potential biases introduced by variable cell-type abundance across developmental stages and species, we additionally performed down-sampling analyses to equalize cell numbers per group before comparing communication scores. For cross-species comparisons, ligand-receptor pairs were mapped to orthologous genes. Communication scores were calculated as the product of averaged ligand expression in sender cells and averaged receptor expression in receiver cells, normalized by group size. We acknowledge that differences in sequencing depth can affect the detection of lowly expressed ligands or receptors; therefore, we excluded pairs with less than 10% detection frequency in either cell type.

## Regulon identification and analysis

Lineage and cell type specific regulon analysis was conducted by SCIENIC in python. Step 1: We used the GRNBoost2 algorithm to construct an initial gene regulatory network (GRN) by inferring transcription factors (TFs)–target gene relationships based on gene co-expression patterns. Step 2: The initial GRN was refined by integrating TF–motif associations and ranking the regulatory potential of each motif for downstream genes. Specifically, motifs were first annotated to their corresponding TFs. Then, based on the ranked list of genes potentially regulated by each motif (by default, the top 5% of all genes), a normalized enrichment score (NES) was calculated using a standardized AUC metric. Motif-gene pairs with NES > 3.0 were retained by default. The network was further pruned using three complementary strategies: thresholds: retaining the top 75% of target genes based on NES ranking for each motif; top_n_targets: retaining the top 50 target genes per TF; top_n_regulators: retaining the top 5 TFs per target gene. The resulting TF-target gene regulatory network is defined as a regulon, in which all retained target genes contain upstream regions enriched for motifs directly bound by the corresponding TF. Step 3: The activity of each regulon was then evaluated across all single cells. This enrichment scoring was performed using a method analogous to the motif enrichment analysis described above. Step 4: To investigate the dynamic regulation of astrocyte subtype developmental lineages, we modeled pseudotemporal trajectories and assessed regulon activity across pseudotime. Linear regression (linregress) was used to evaluate differences in regulon dynamics between lineages or cell types. Regulons with P-value < 0.05 and an effect size f² > 0.2 were considered significant.

**Supplementary Table S1.** Stage definition in this study

| Stage | Age |
| --- | --- |
| Late mid-fetal | 19 PCW ≤ Age < 24 PCW |
| Late fetal | 24 PCW ≤ Age <38 PCW |
| Neonatal | 0 M (birth) ≤ Age <2M |
| Infancy | 2M ≤ Age <12M |
| Early childhood | 1 Y ≤ Age <6 Y |
| Late childhood | 6 Y ≤ Age < 12 Y |
| Late childhood | 12 Y ≤ Age < 20 Y |
| Adolescence | 20 Y ≤ Age < 40 |
| Young adulthood | 40 Y ≤ Age < 60 Y |

PCW: Post-conceptional week; M: Month; Y: Year

**Supplementary Table S3.** Stability assessment of human brain astrocyte subtypes based on Jaccard similarity analysis

| Subtypes | Boot Mean | Subset Mean | Noise Mean | Bojit Mean | Jitter Mean |
| --- | --- | --- | --- | --- | --- |
| iAstro | 0.634 | 0.604 | 0.640 | 0.613 | 0.645 |
| fAstro | 0.603 | 0.601 | 0.629 | 0.608 | 0.613 |
| pAstro | 0.881 | 0.902 | 0.895 | 0.901 | 0.899 |

Mean: average Jaccard similarity via 100 iterations of subsampling. Mean Jaccard Similarity>0.85, Highly stable / Robust; Mean Jaccard Similarity 0.6-0.75, Doubtful / Uncertain assignment; Mean Jaccard Similarity<0.6, Untrustworthy.

**Supplementary Table S4.** Astrocyte-associated synaptic development and related genes

| Biological process | Genes |
| --- | --- |
| Synaptogenesis | THBS1, THBS2, THBS3, THBS4, GPC4, GPC6, PTPRD, PTPX3, NRCAM, HEPACAM, EFNA3, EPHA4, GJB6, BDNF, WNT2, WNT5A |
| Synaptic maturation | SPARCL1, CHRDL1, SERBP, IL10, IL6, HMGCR, APOE, TNF, SLC1A2, SLC1A3, KCNJ10, IGF1 |
| Synaptic pruning | MEGF10, MERTK, C1QA, C3, C4, CRKII, DOCK180, ABCA1 |

**Supplementary Table S5.** List of abbreviations used in the manuscript.

| Abbreviations | Full terms |
| --- | --- |
| PCW | Post-conceptional week |
| PFC | Prefrontal cortex |
| iAstro | Interlaminar astrocyte |
| l1Astro | Layer 1 astrocyte |
| pAstro | Protoplasmic astrocyte |
| fAstro | Fibrous astrocyte |
| vAstro | Varicose projection astrocytes |
| DEGs | Differentially expressed genes |
| DEPs | Differentially expressed proteins |
| ExN | Excitatory neurons |
| InN | Inhibitory neurons |
| GSA | Genome sequence archive |
| GO | Gene ontology |
| BP | Biological process |
| PEM | Principal eigenvalue matrix |
| TFs | Transcription factors |

# Supplementary Figures


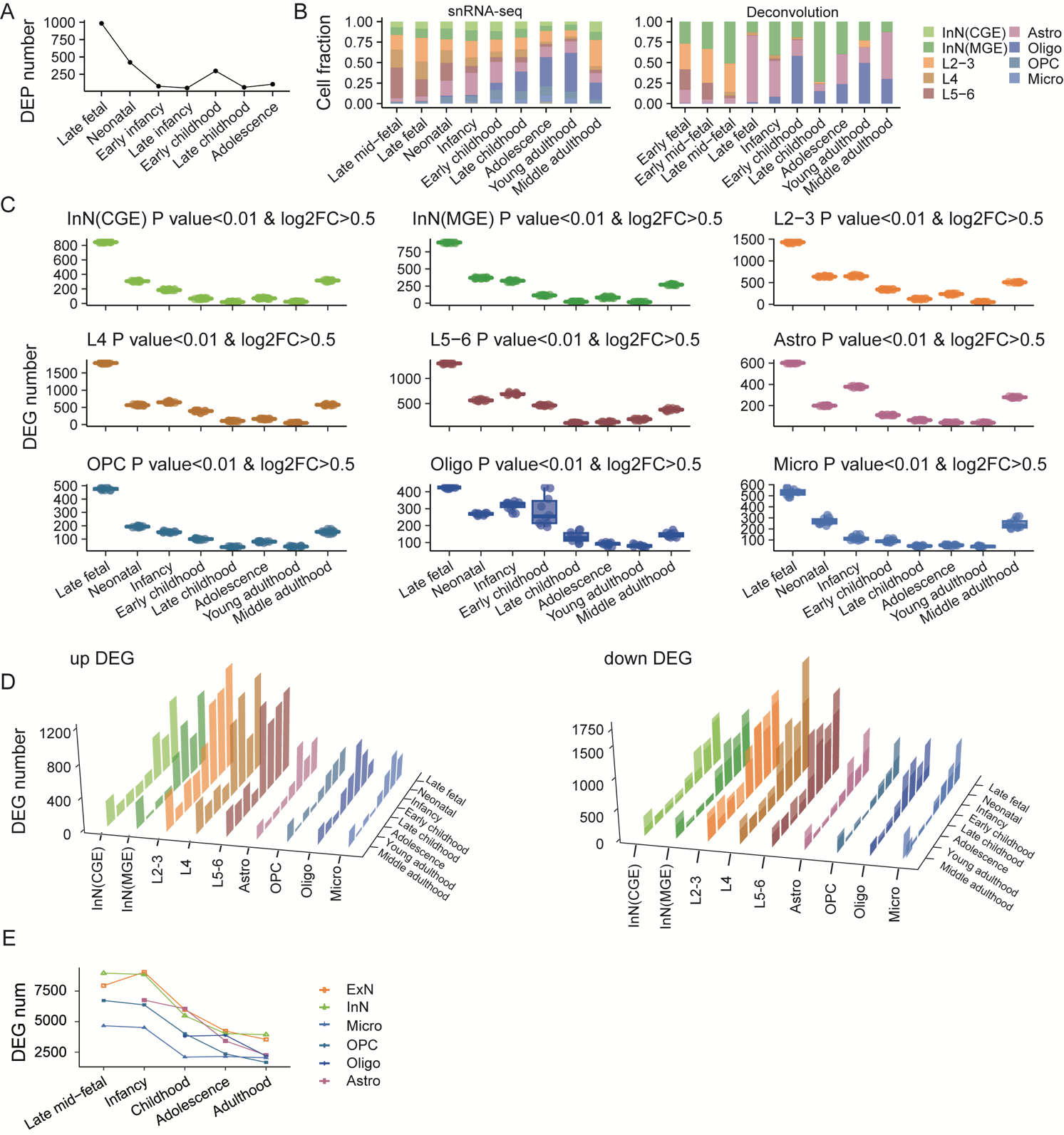


**Fig. S1.** Dynamic transcriptomic change, proteomic change and cellular composition during human brain development. A. Number of differentially expressed proteins (DEPs) between adjacent developmental stages during human cortical development. B. Cellular composition across different stages of human brain development. The left panel shows cell proportion distributions from scRNA-seq data, while the right panel depicts cell composition after tissue-level deconvolution. InN (CGE): Inhibitory neurons from caudal ganglionic eminence; InN (MGE): Inhibitory neurons from medial ganglionic eminence. L2-3: Excitatory neurons in layer 2 and layer3; L4: Excitatory neurons in layer 4; L5-6: Excitatory neurons in layer 5 and layer6; Astro: Astrocytes; OPC: Oligodendrocyte precursor cells; Oligo: Oligodendrocytes; Micro: Microglia. C. Distribution of differentially expressed genes (DEGs) between consecutive developmental stages after down-sampling each stage to the same number of cells, repeated ten times. D. Number of up and down DEGs for each cell type during human brain development. E. Number of DEGs for each cell type during human brain development in the dataset from Zhu et al.


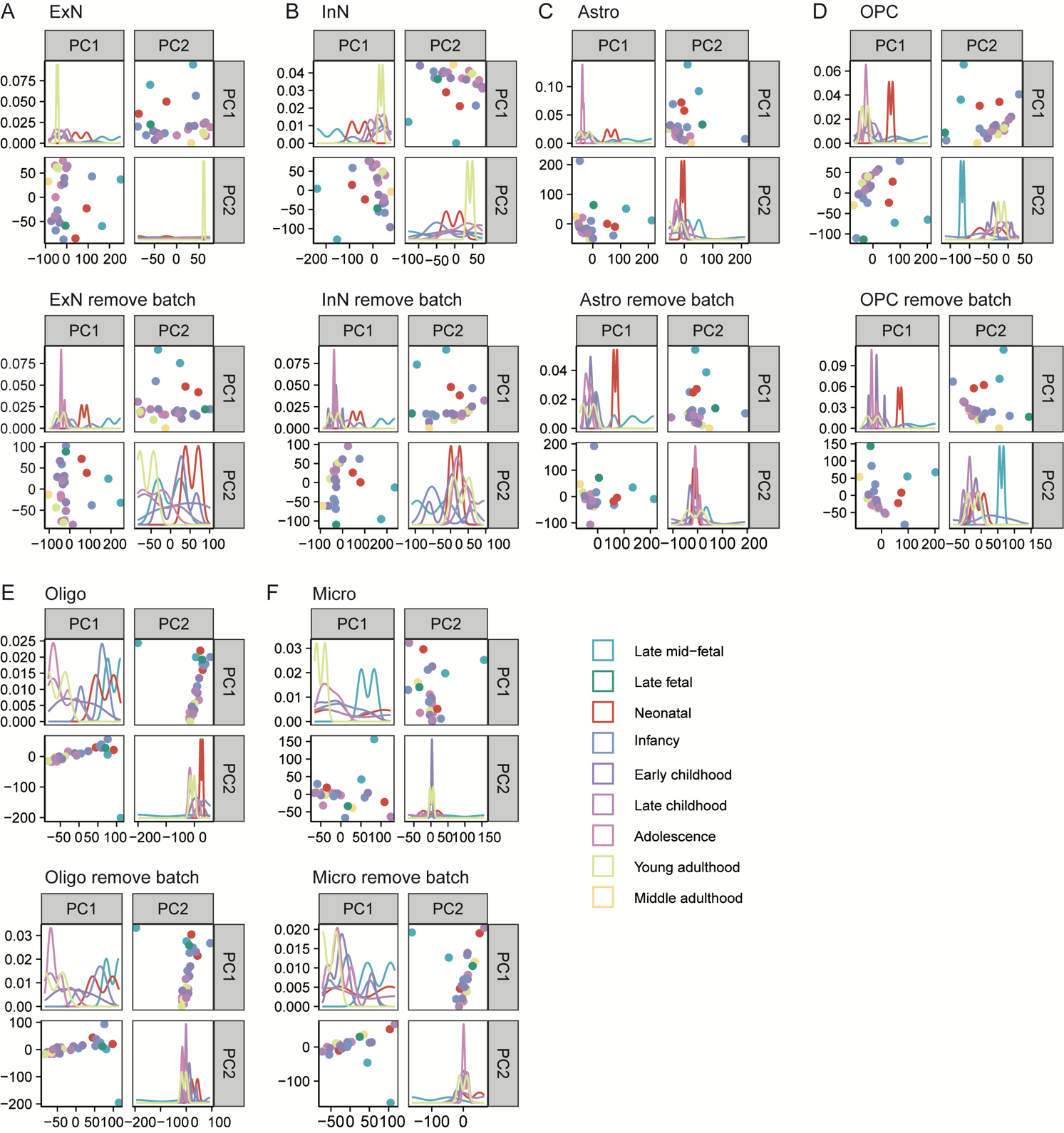


**Fig. S2.** A-F. PCA distribution of each cell type before and after batch effect correction.


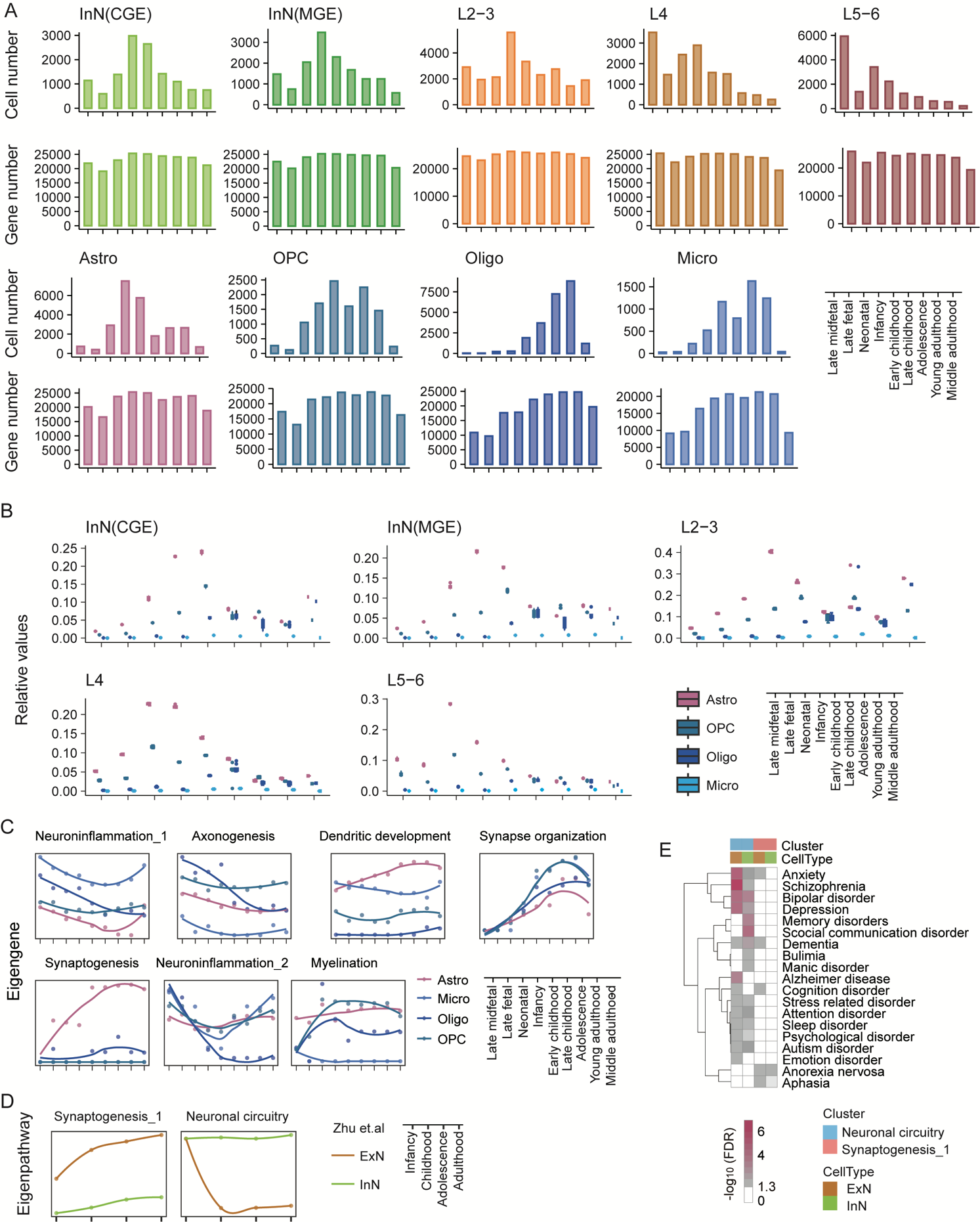


**Fig. S3.** Cell quality control and glia-neuron communication. A. Cell counts and the number of expressed genes across developmental stages and cell types. B. Dynamic signaling values from down-sampling analysis of glia-neuron communication. Relative values on the y-axis represent glia-cell communication strength, with higher values indicating stronger communication. C. Glia-neuron signaling pathways were grouped into seven clusters based on their temporal activity patterns, associated with neuroninflammation, axonogenesis, dendritic development, synapse organization, synaptogenesis, and myelination. The y-axis indicates the relative activity of each pathway, derived from principal component analysis of pathway activity. Higher values reflect greater pathway activity relative to other cells or developmental stages. D. Astrocyte-neuron signaling pathways associated with neuronal circuitry and synaptogenesis in the dataset from Zhu et al. The y-axis indicates the relative activity of each pathway, derived from principal component analysis of pathway activity. Higher values reflect greater pathway activity relative to other cells or developmental stages. E. Enrichment of genes related to neuronal circuitry and synaptogenesis in psychiatric and neurological disorders from the GWAS Catalog database. Enrichment was assessed using Fisher’s exact test, and P values were adjusted for multiple testing using the Benjamini-Hochberg method (FDR < 0.05).


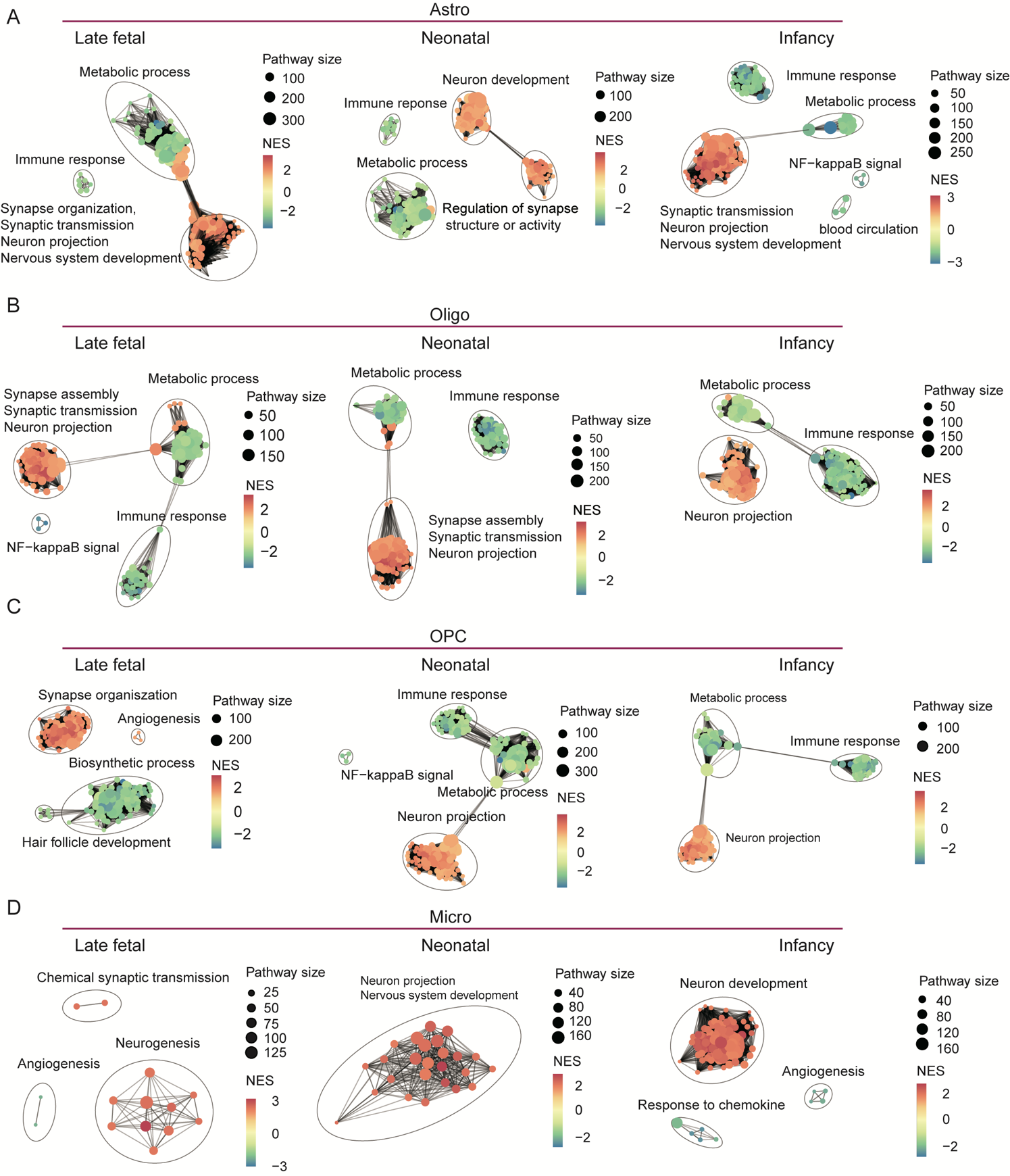


**Fig. S4.** Functional enrichment analysis of biological processes involving ligands and receptors in glia-neuron communication during periods of pronounced transcriptomic change.


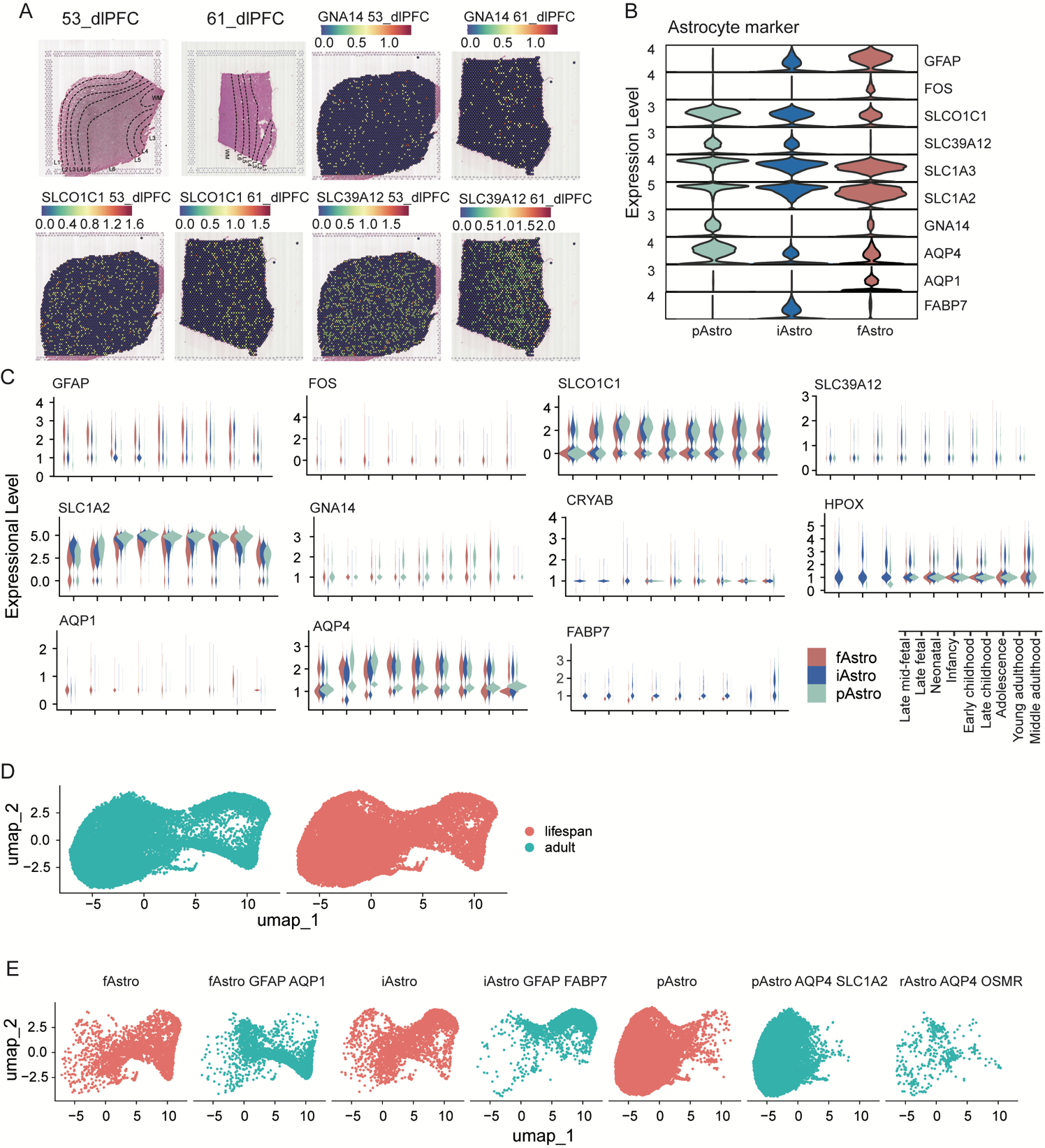


**Fig. S5.** Expression of astrocyte subtype markers and results of integration analyses. A. Expression of astrocyte subtype markers in the human spatial transcriptome. B. Expression of astrocyte subtype markers across all developmental stages. C. Expression patterns of astrocyte subtype marker genes across developmental stages. D. Integration of human lifespan astrocyte scRNA-seq data from the Lister lab with adult human brain data from the Sestan lab. E. UMAP distribution of astrocyte subtypes across the two datasets following data integration.


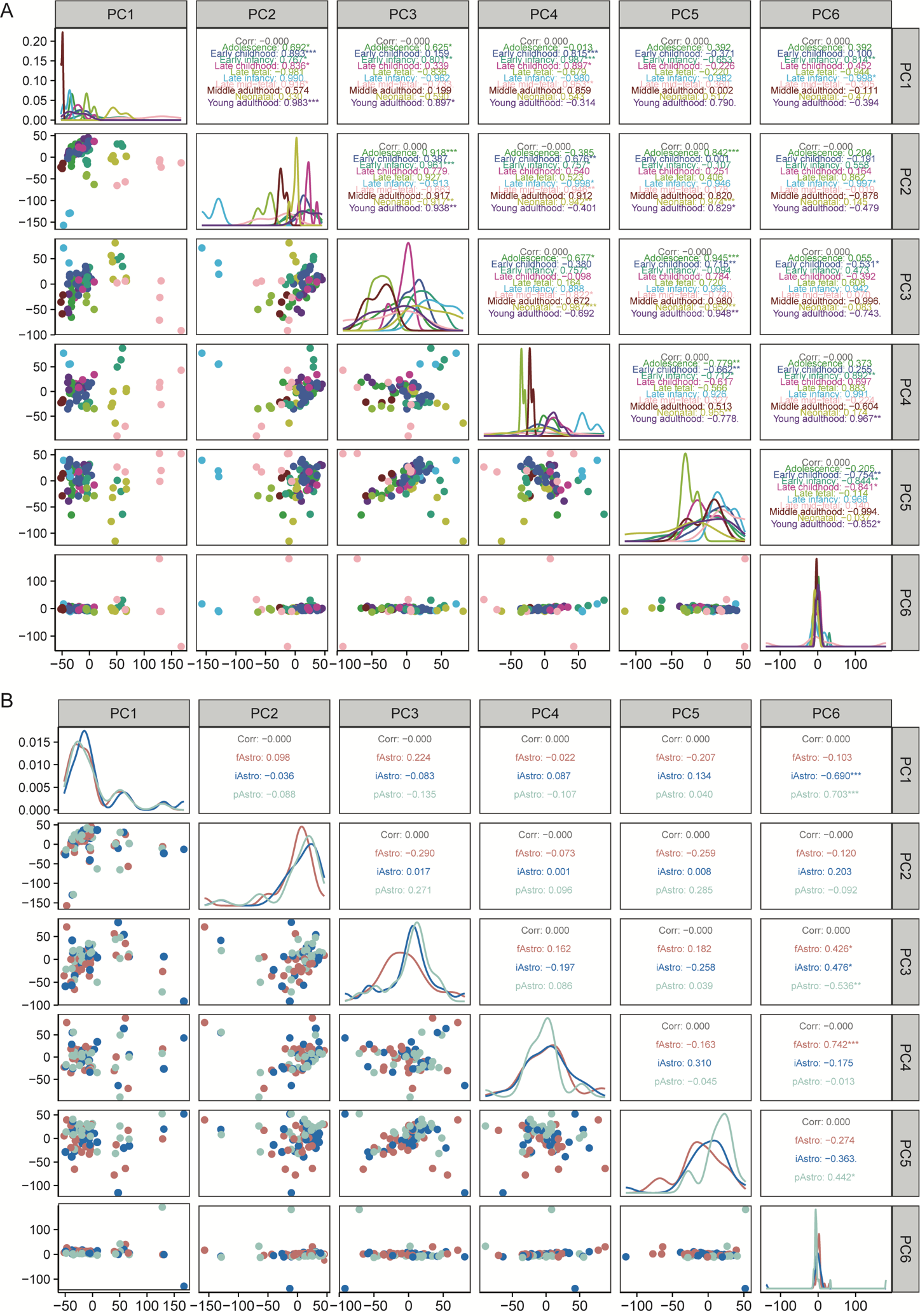


**Fig. S6.** PCA distribution of astrocyte subtypes. A. PCA distribution of astrocytes during human brain development, emphasizing the distribution of samples for the first six PCs. In the panel, 'corr' represents the correlation coefficient, where a value greater than 0 indicates a positive correlation between this astrocyte subtype and other astrocyte subtypes, with larger values reflecting a stronger positive correlation. Conversely, a value less than 0 indicates a negative correlation between this astrocyte subtype and other subtypes, with smaller values representing a stronger negative correlation. B. PCA distribution of astrocyte subtypes during human brain development, highlighting the sample distribution for the first six PCs.


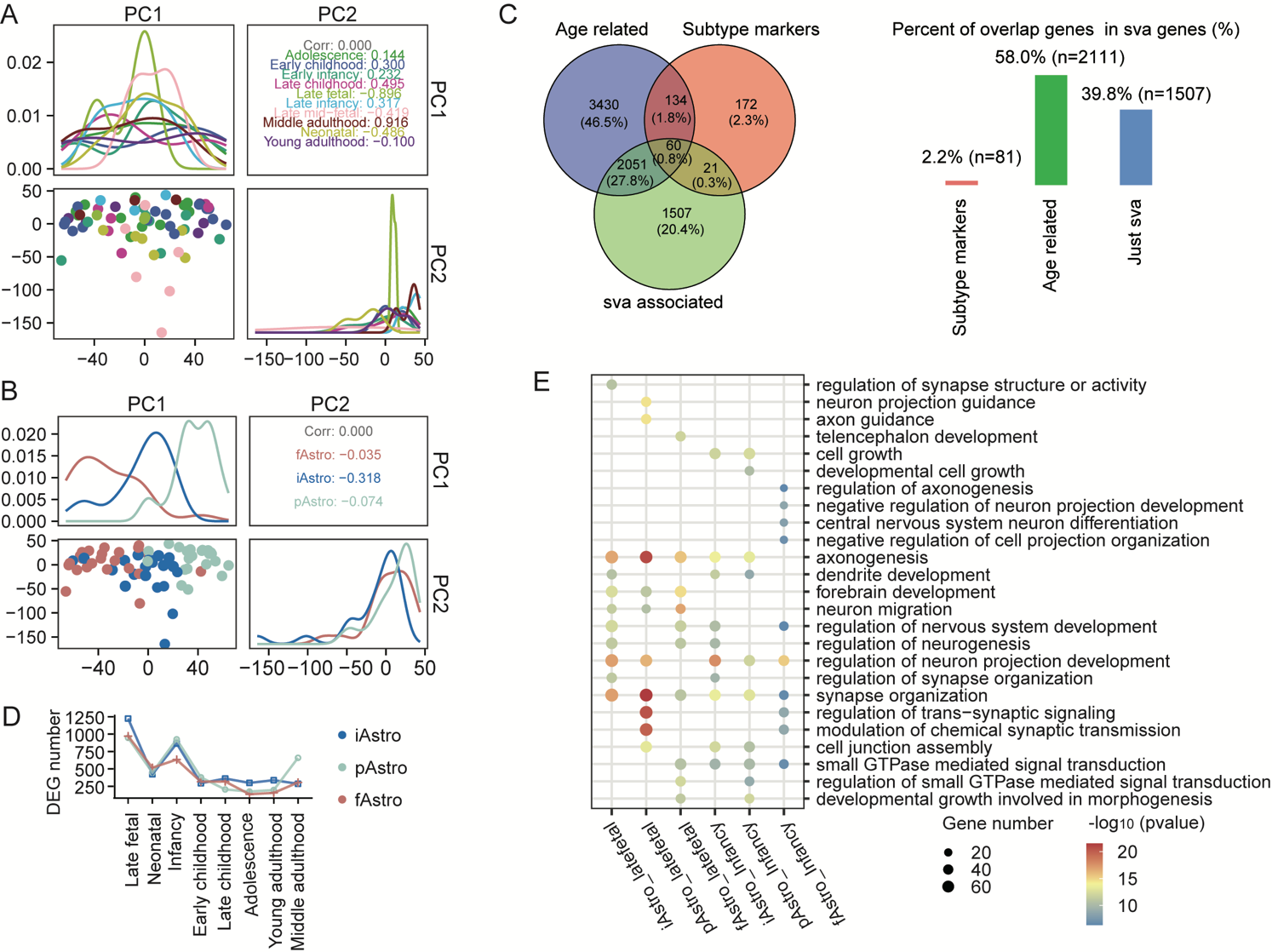


**Fig. S7.** Transcriptomic heterogeneity of astrocyte subtypes. A. PCA distribution of astrocytes during human brain development after removing age effects, highlighting sample distribution along the first two principal components PCs. B. PCA distribution of astrocyte subtypes during human brain development after removing age effects, highlighting sample distribution along the first two principal components PCs. C. Intersection analysis of age-associated genes, astrocyte subtype markers, and genes related to age effects after adjustment using sva, to further explore their relationships. E. overlapping genes within the gene set obtained after removing age effects via sva. D. Distribution of DEGs across astrocyte subtypes between consecutive developmental stages. E. Biological process enrichment of DEGs in fetal and infancy stages for astrocyte subtypes. All terms met the significance threshold following Benjamini-Hochberg correction (false discovery rate, FDR < 0.05).

_­­_
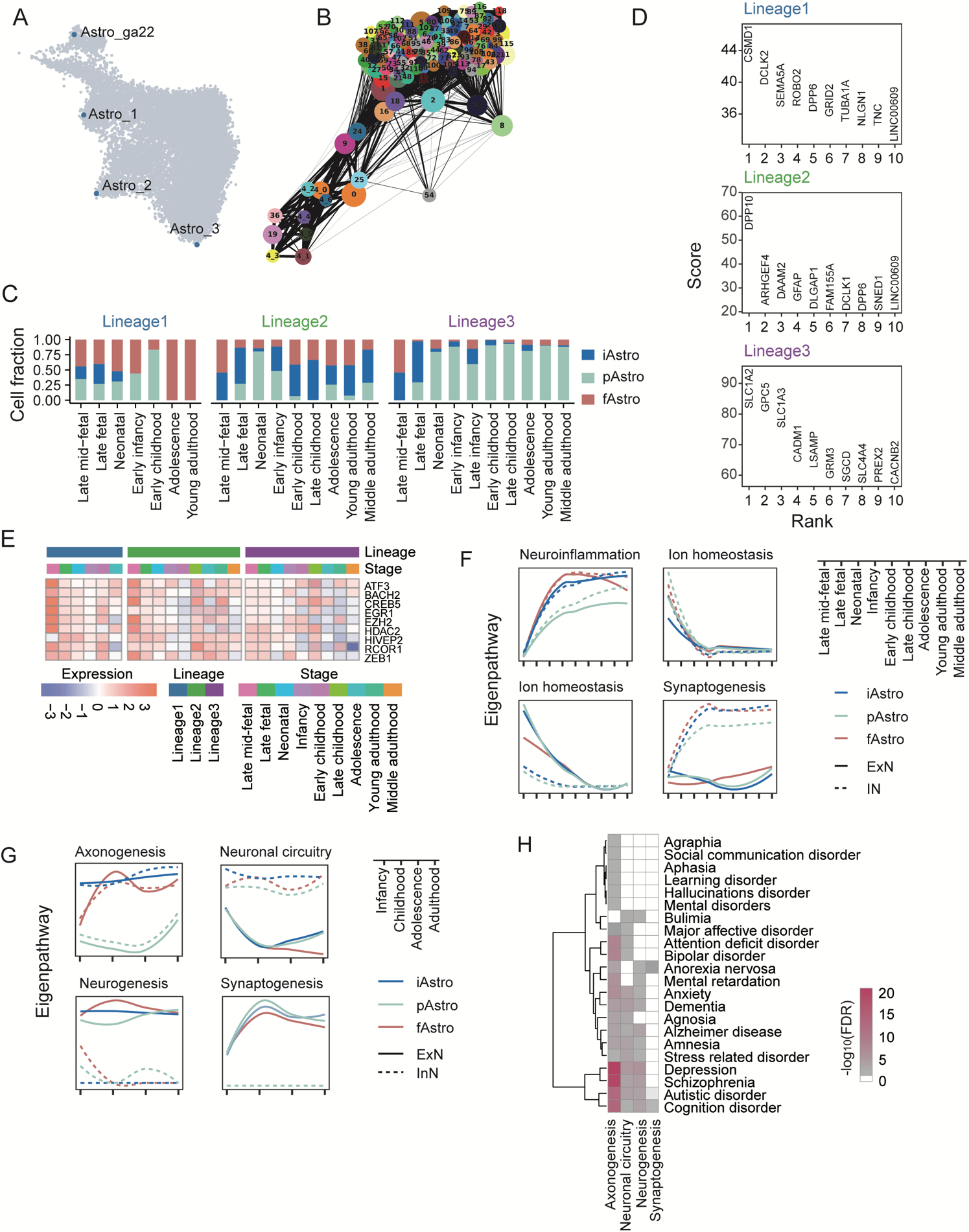


**Fig. S8.** Developmental trajectory of astrocytes and astrocyte-neuron communication. A. Start and end points of the astrocyte developmental lineage identified using the PAGA package. B. Linkage degree among cell clusters and UMAP plot displaying these clusters. C. Distribution of cell fractions across each astrocyte developmental lineage. D. Marker genes ranked by expression for each astrocyte developmental lineage. E. Dynamic transcriptional regulatory activity of transcription factors (TFs) within astrocyte subtype lineage- conserved regulons. The regulatory activity value of each TF was averaged across cells within each developmental stage, and the resulting values were subsequently z-normalized. F. Four distinct communication patterns of astrocyte subtype-neuron signaling pathways, associated with neuroinflammation, ion homeostasis, and synaptogenesis. The y-axis indicates the relative activity of each pathway, derived from principal component analysis of pathway activity. Higher values reflect greater pathway activity relative to other cells or developmental stages. G. Astrocyte-neuron signaling pathways associated with neurogenesis, axonogenesis, neuronal circuitry and synaptogenesis in the dataset from Zhu et al. The y-axis indicates the relative activity of each pathway, derived from principal component analysis of pathway activity. Higher values reflect greater pathway activity relative to other cells or developmental stages. H. Enrichment of genes related to axonogenesis, neuronal circuitry, neurogenesis, and synaptogenesis in psychiatric and neurological disorders from the GWAS Catalog database. Enrichment was assessed using Fisher’s exact test, and P values were adjusted for multiple testing using the Benjamini-Hochberg method (FDR < 0.05).


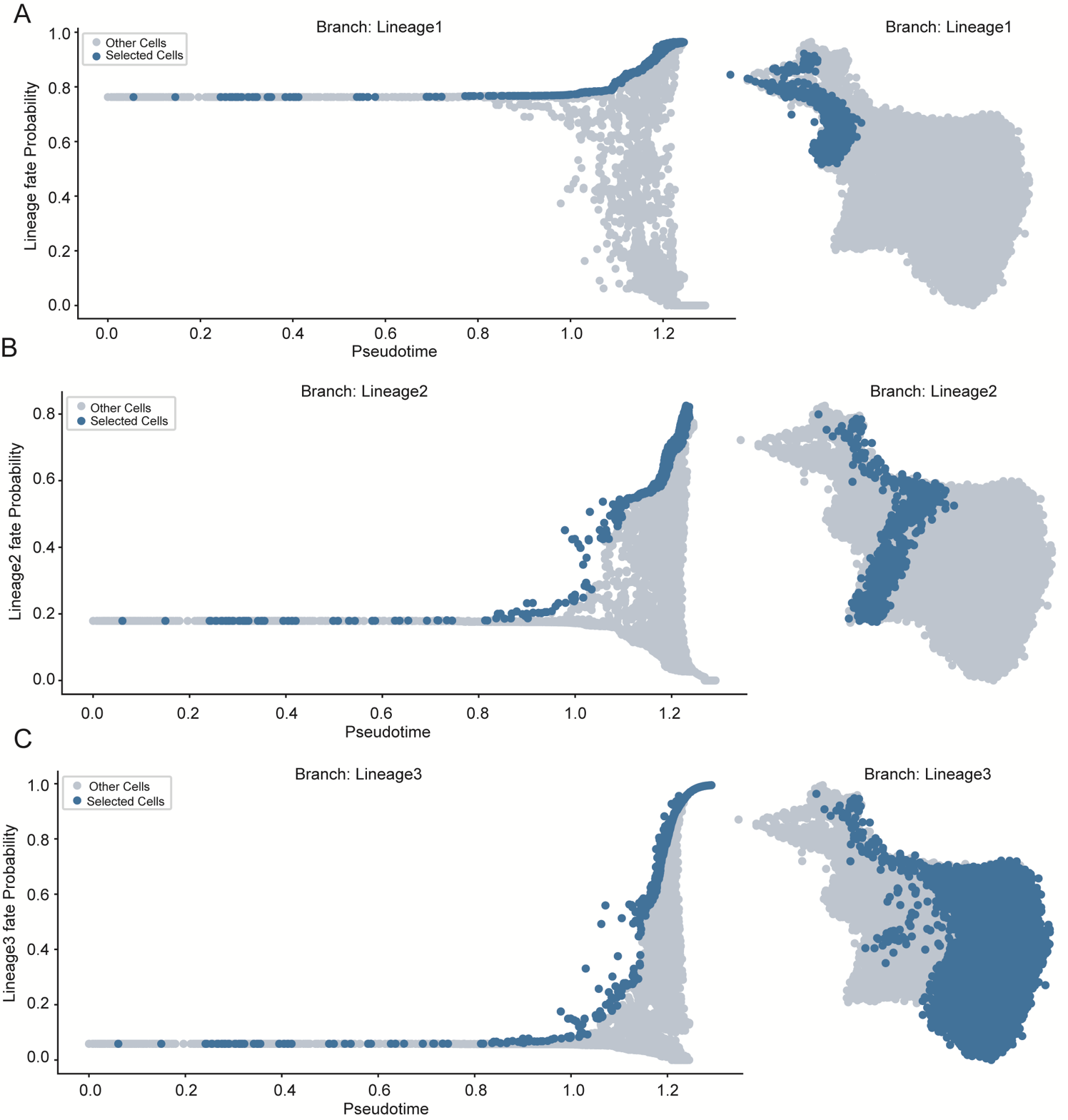


**Fig. S9.** Trajectory of astrocyte subtypes during human brain development. A-C. Developmental trajectories of Lineage 1 to Lineage 3.


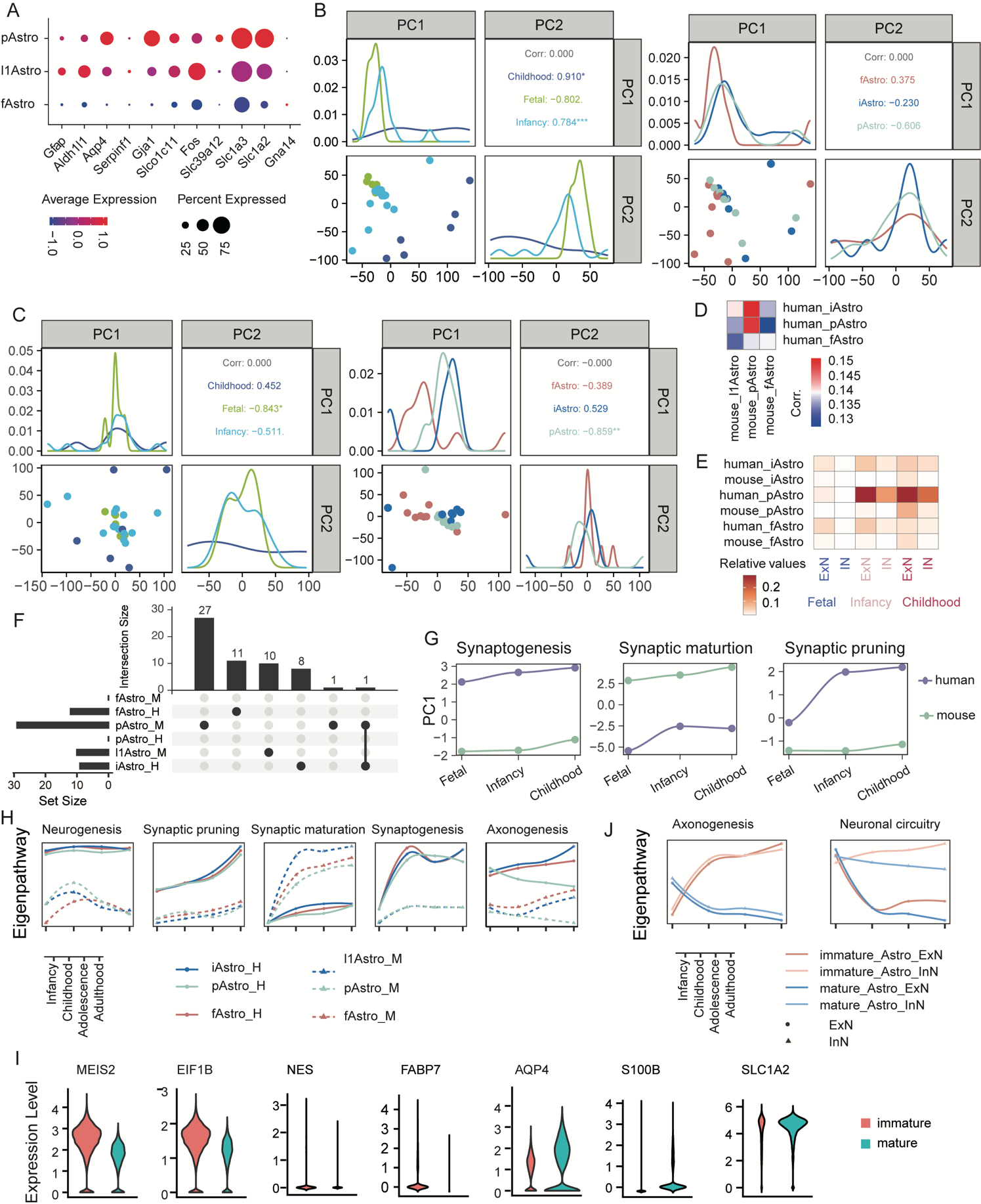


**Fig. S10.** Comparison of astrocyte subtypes between the human and mouse brains. A. Expression of astrocyte subtype markers in the mouse brain. B. PCA distribution of astrocytes during mouse brain development before removing age effects, highlighting sample distribution along the first two principal components PCs of development stages and astrocyte subtypes. C. PCA distribution of astrocytes during mouse brain development after removing age effects, highlighting sample distribution along the first two principal components PCs of development stages and astrocyte subtypes. D. Correlation between human and mouse astrocyte subtypes assessed by Pearson correlation (p < 0.05). Corr.: Pearson correlation coefficient. E. Comparison of astrocyte subtype-neuron signaling between the human and mouse brain. F. Upset plot depicting shared TFs in human and mouse astrocyte subtype-specific regulons, using datasets from the human cerebral cortex and mouse visual cortex. iAstro_H, human interlaminar astrocytes; pAstro_H, human protoplasmic astrocytes; fAstro_H, human fibrous astrocytes. l1Astro_M, mouse astrocytes locating in layer 1; pAstro_M, mouse protoplasmic astrocytes; fAstro_M, mouse fibrous astrocytes. G. Analysis of expression differences in genes regulating synaptogenesis, synaptic maturation and synaptic pruning by astrocytes in human and mouse brains. H. Astrocyte-neuron communication in human and mouse brains associated with neurogenesis, synaptic pruning, synaptic maturation, synaptogenesis, and axonogenesis in the dataset from Gao et al. The y-axis indicates the relative activity of each pathway, derived from principal component analysis of pathway activity. Higher values reflect greater pathway activity relative to other cells or developmental stages. H. Expression of marker genes in immature and mature astrocytes. I. Marker gene expression distinguishing immature and mature astrocytes. J. Astrocyte-neuron signaling pathways associated with axonogenesis and neuronal circuitry in the dataset from Zhu et al. The y-axis indicates the relative activity of each pathway, derived from principal component analysis of pathway activity. Higher values reflect greater pathway activity relative to other cells or developmental stages.


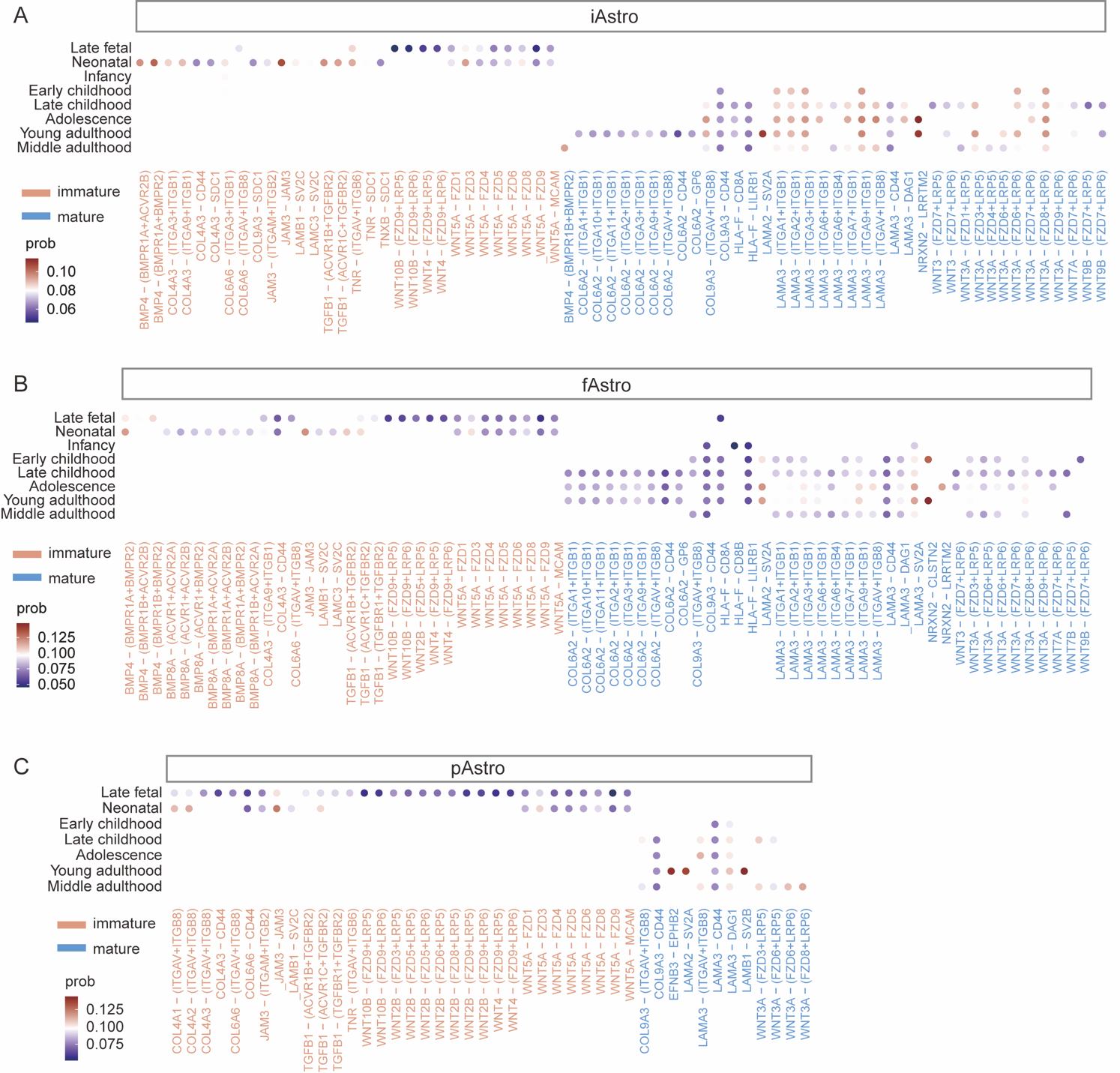


**Fig. S11.** Ligand-receptor pairs involved in axonogenesis. A-C. Immature and mature astrocytes subtypes enriched ligand-receptor pairs when comparing these two cell states. prob, interaction probability.
