# Supplementary figures and images for: The Role of Astrocyte–Neuron Interactions in Shaping Neuronal Maturation during Human Brain Development
Source: Comput Struct Biotechnol J. 2026 May 18;2026issue-1(1):0083. doi: 10.34133/csbj.0083 (PMC13181173; doi:10.34133/csbj.0083)

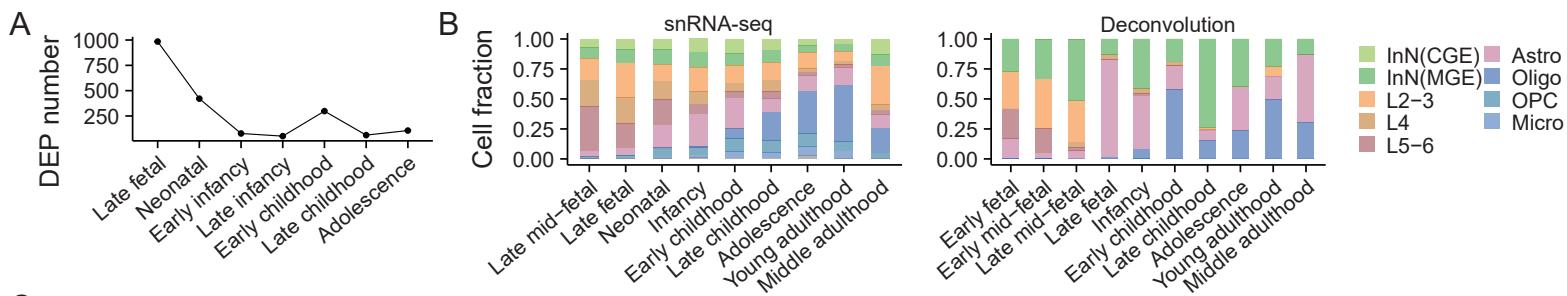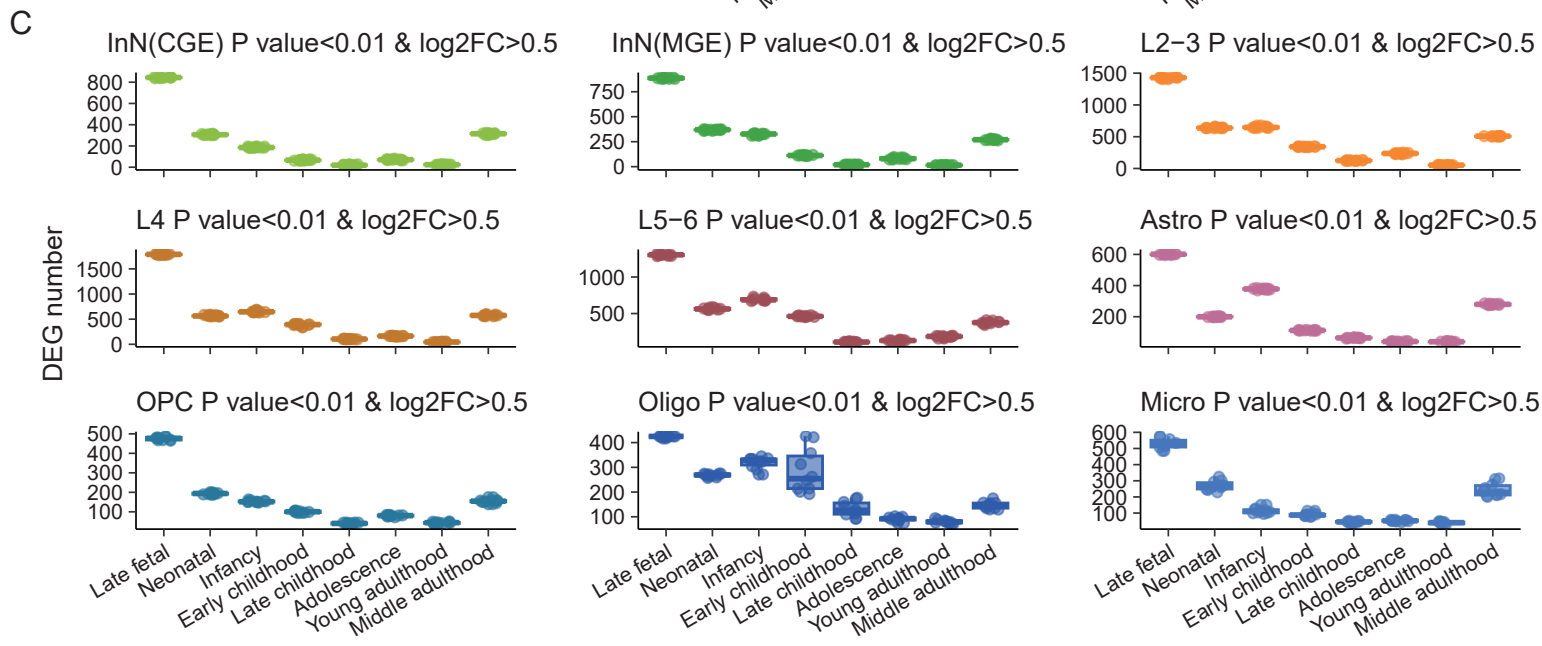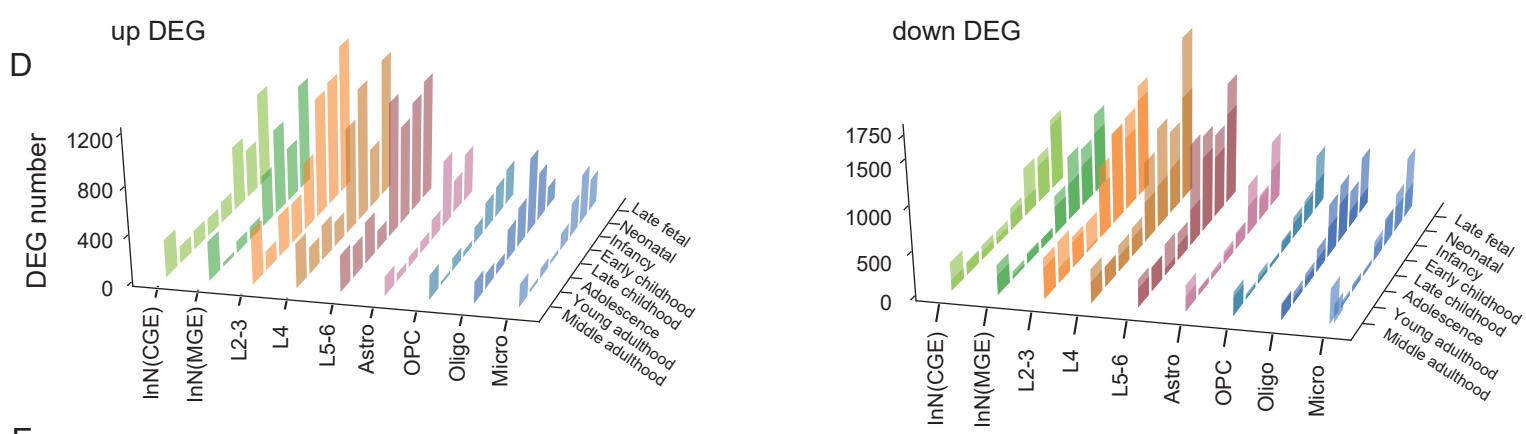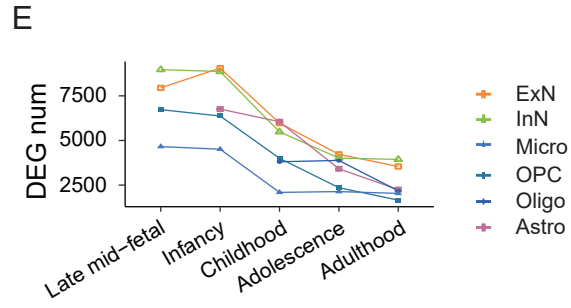

Supplement: Supplementary 1 — Supplementary Text Tables S1 to S5 Figs. S1 to S11 [file csbj.0083.f1.zip › FigS1.pdf]

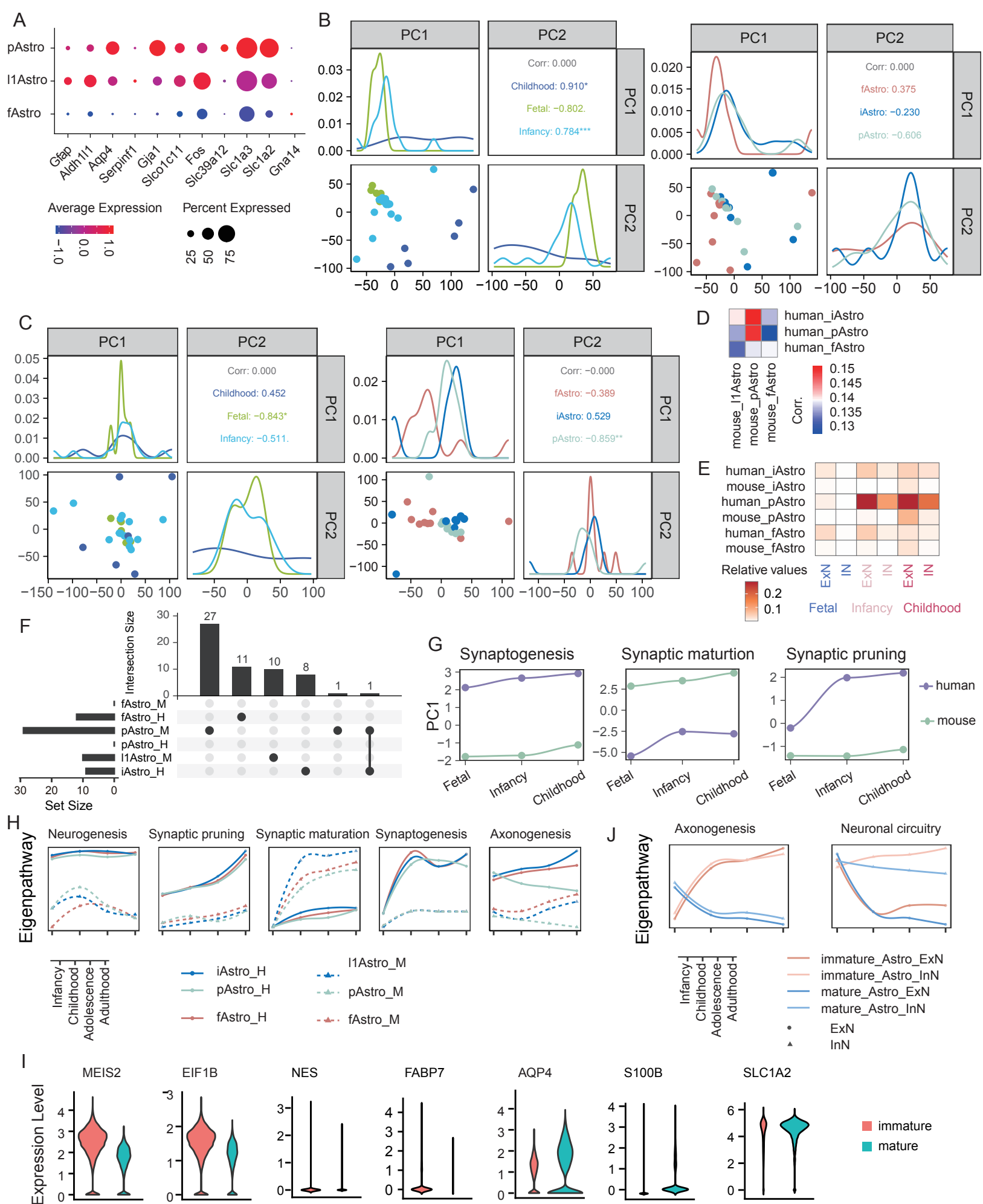

Supplement: Supplementary 1 — Supplementary Text Tables S1 to S5 Figs. S1 to S11 [file csbj.0083.f1.zip › FigS10.pdf]

A

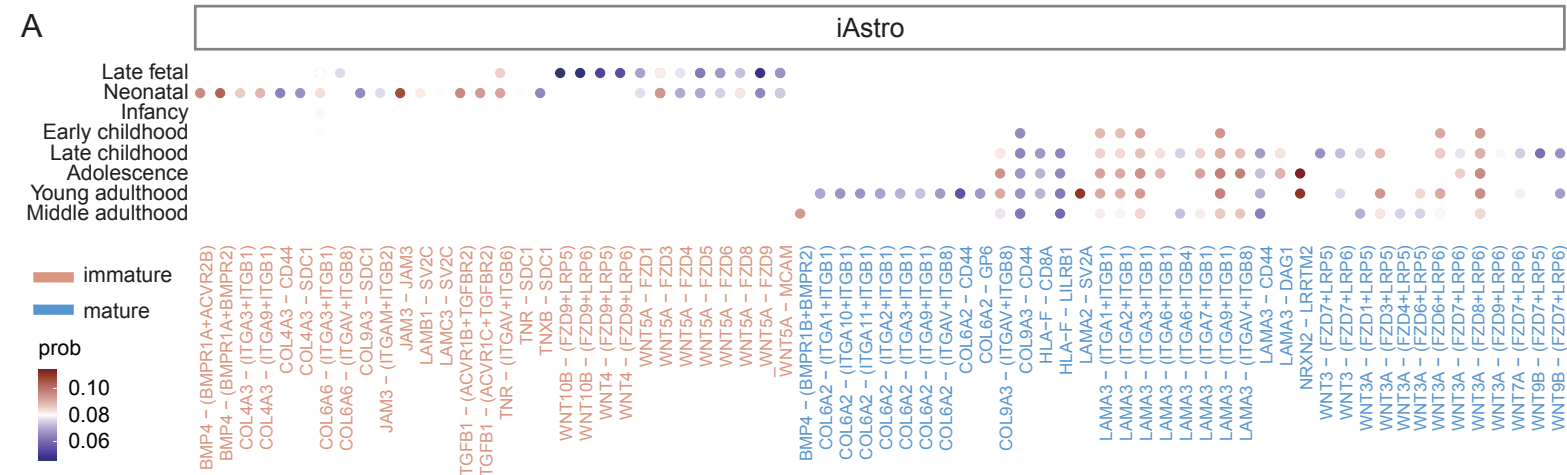

B

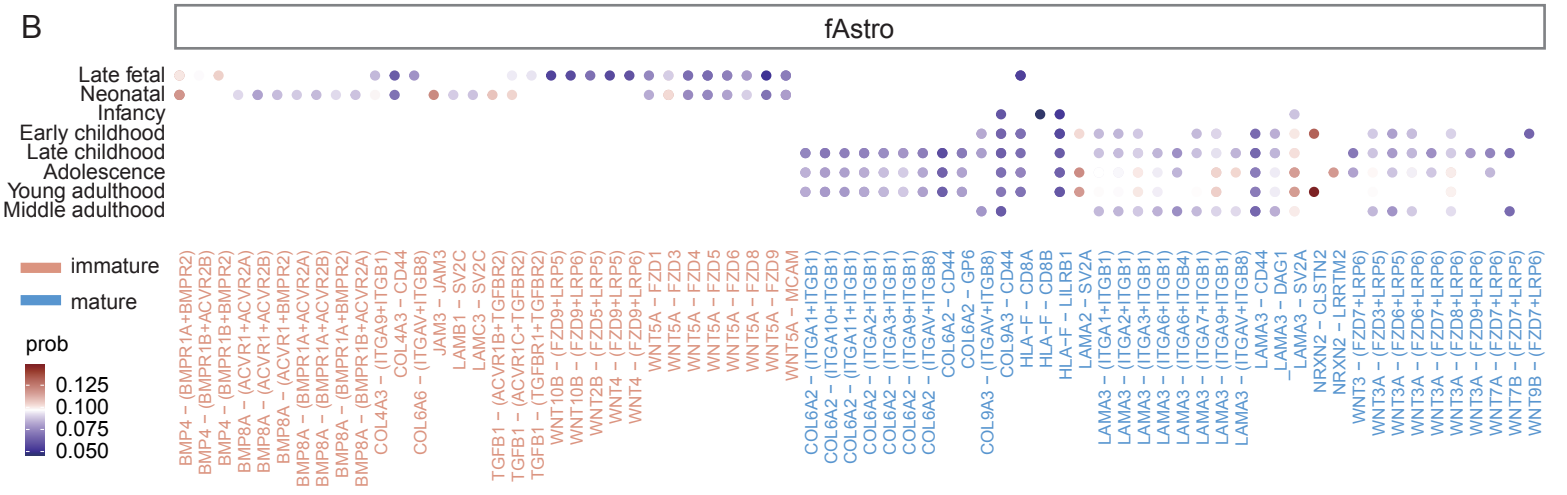

C

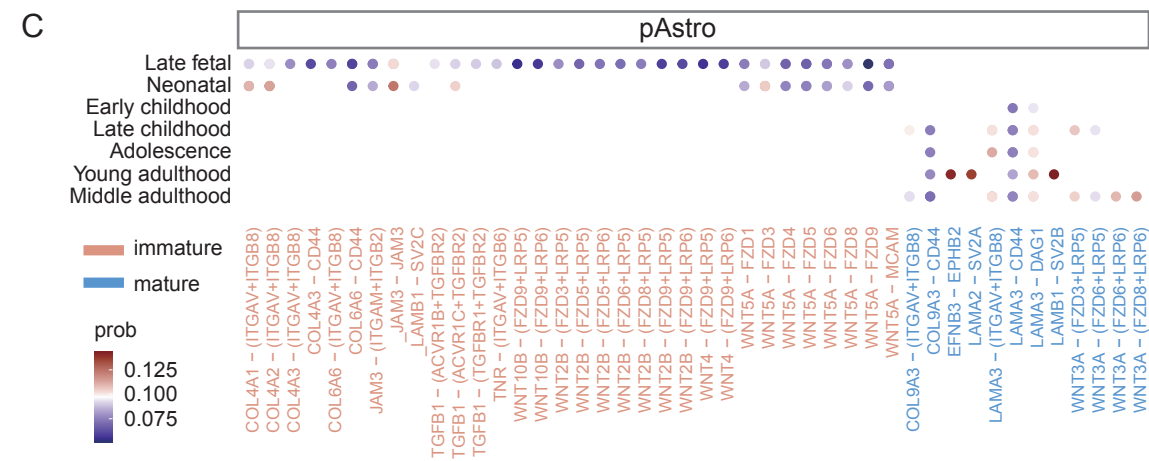

Supplement: Supplementary 1 — Supplementary Text Tables S1 to S5 Figs. S1 to S11 [file csbj.0083.f1.zip › FigS11.pdf]

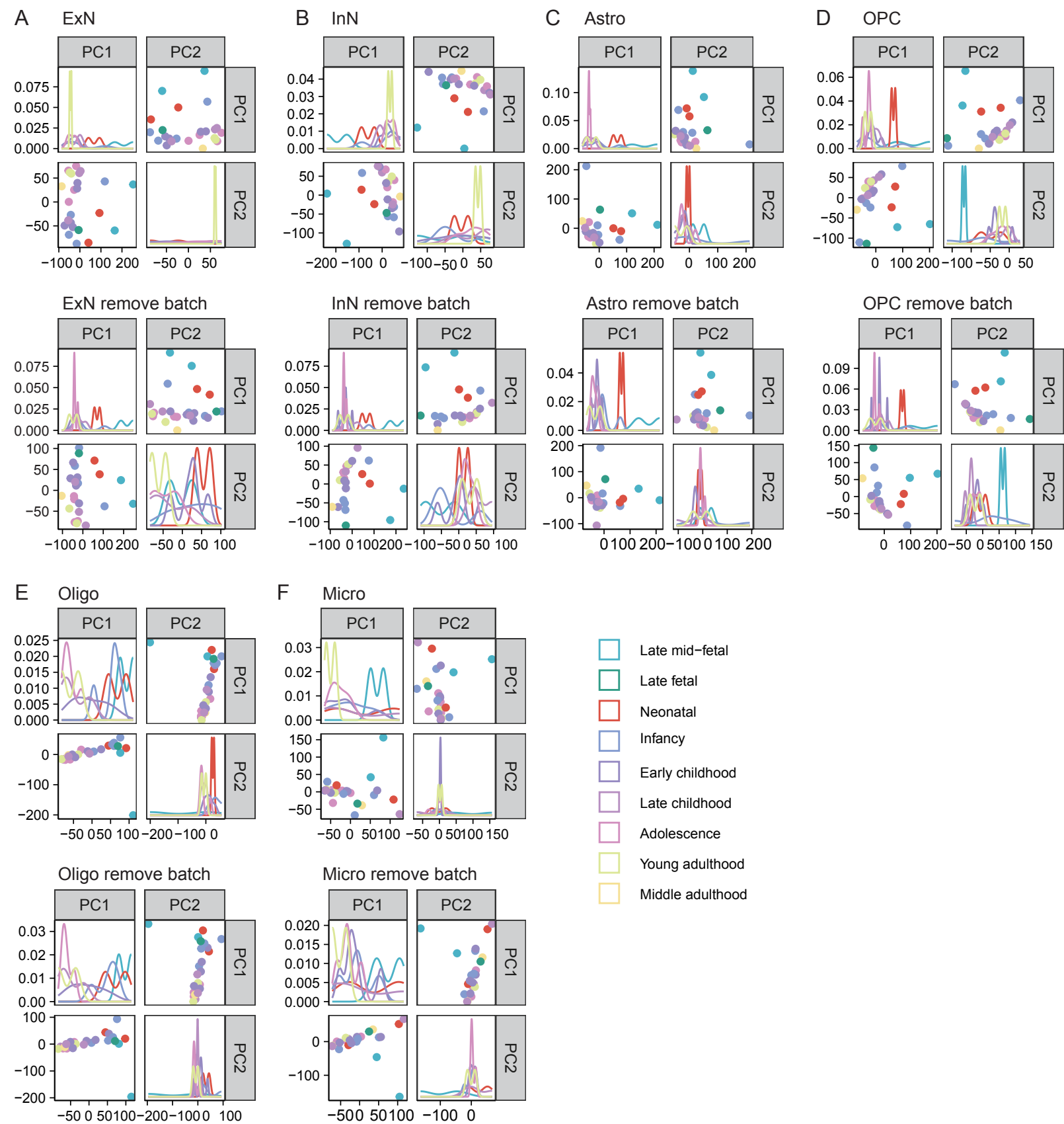

Supplement: Supplementary 1 — Supplementary Text Tables S1 to S5 Figs. S1 to S11 [file csbj.0083.f1.zip › FigS2.pdf]

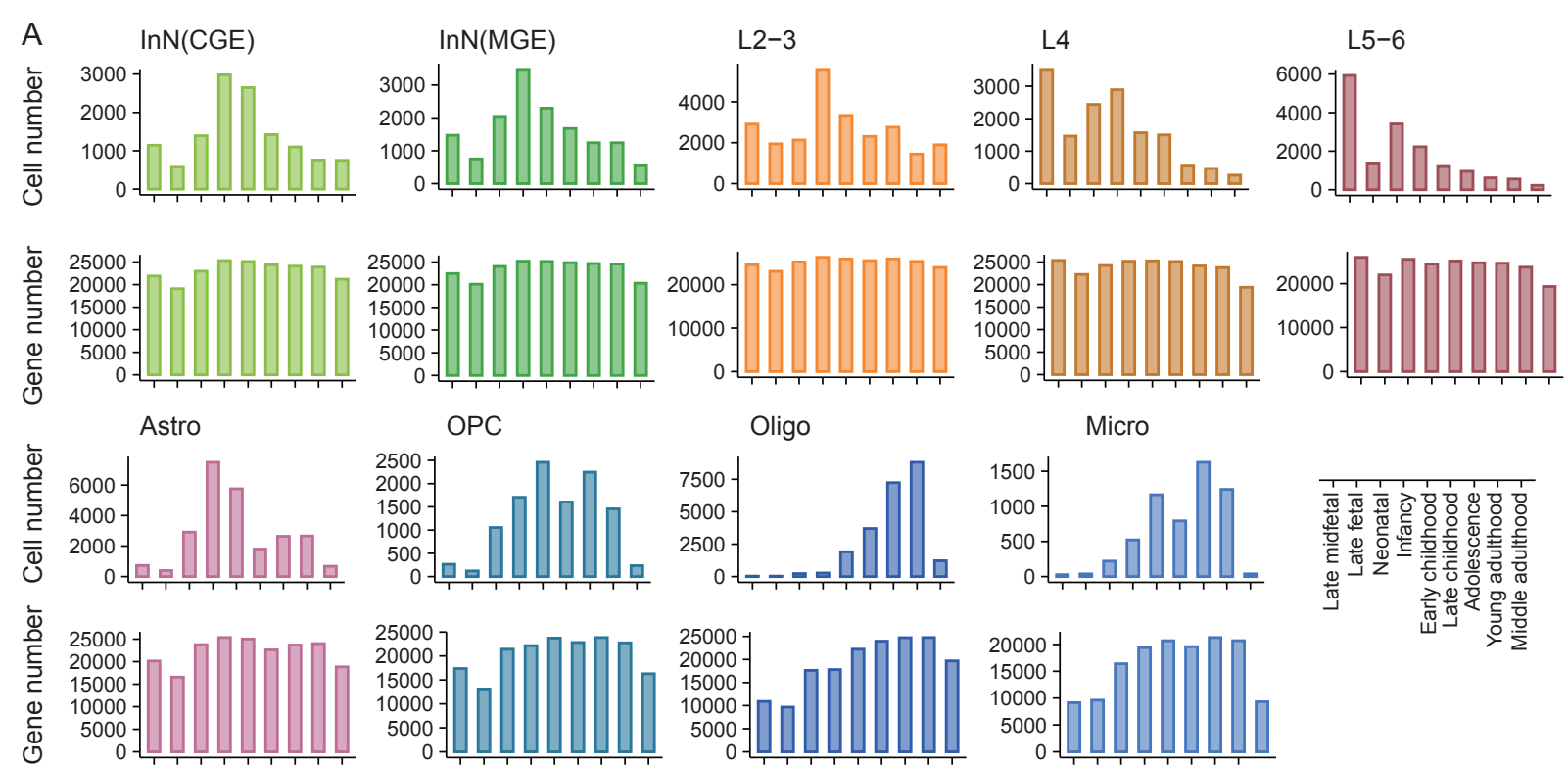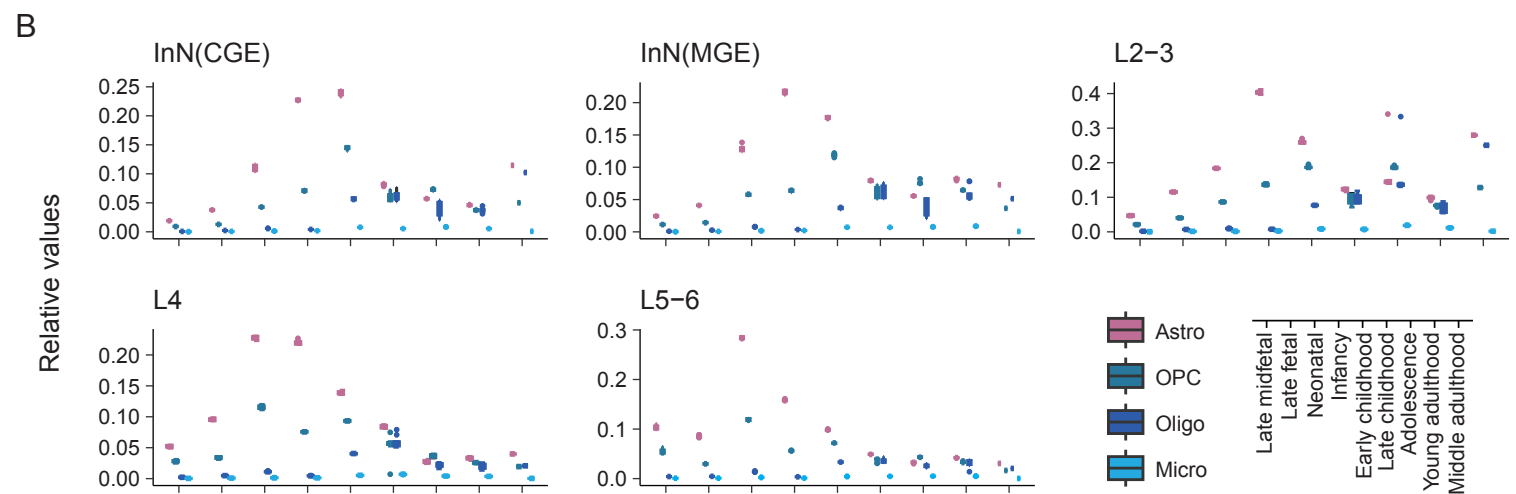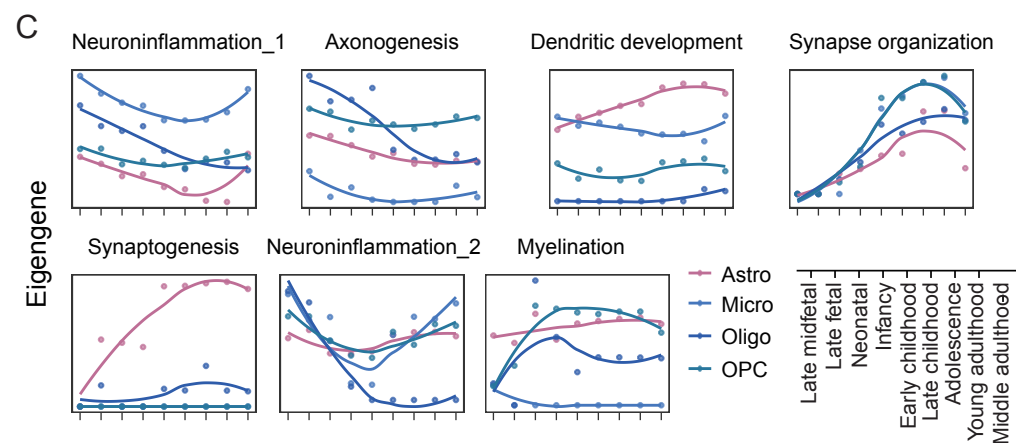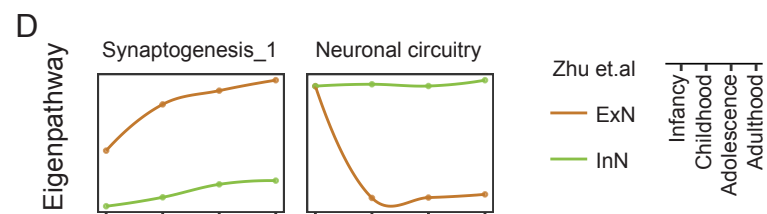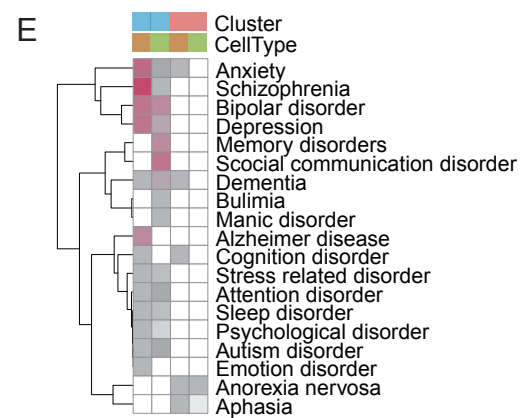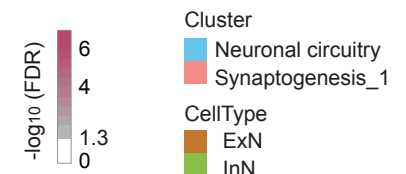

Supplement: Supplementary 1 — Supplementary Text Tables S1 to S5 Figs. S1 to S11 [file csbj.0083.f1.zip › FigS3.pdf]

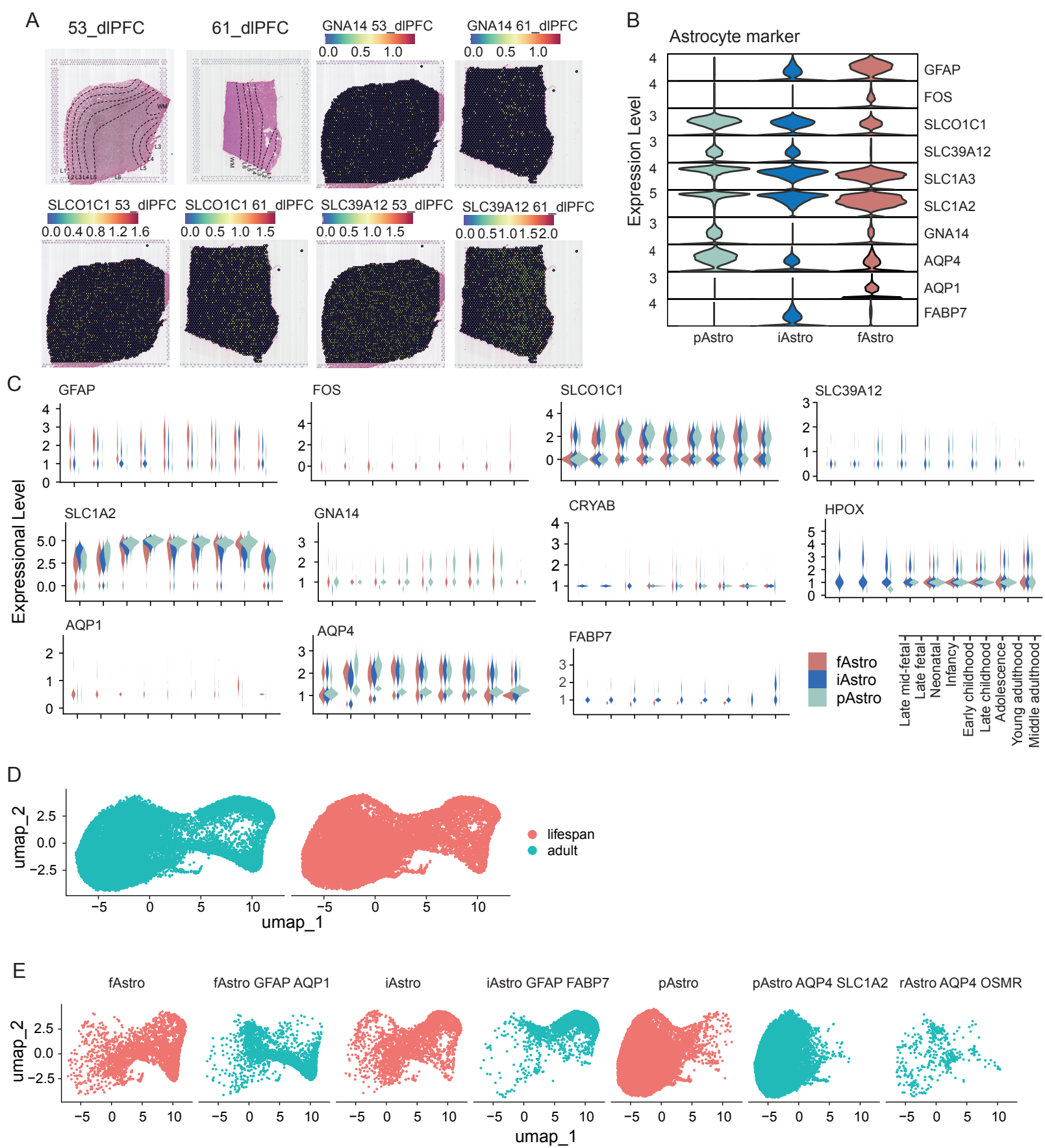

Supplement: Supplementary 1 — Supplementary Text Tables S1 to S5 Figs. S1 to S11 [file csbj.0083.f1.zip › FigS5.pdf]

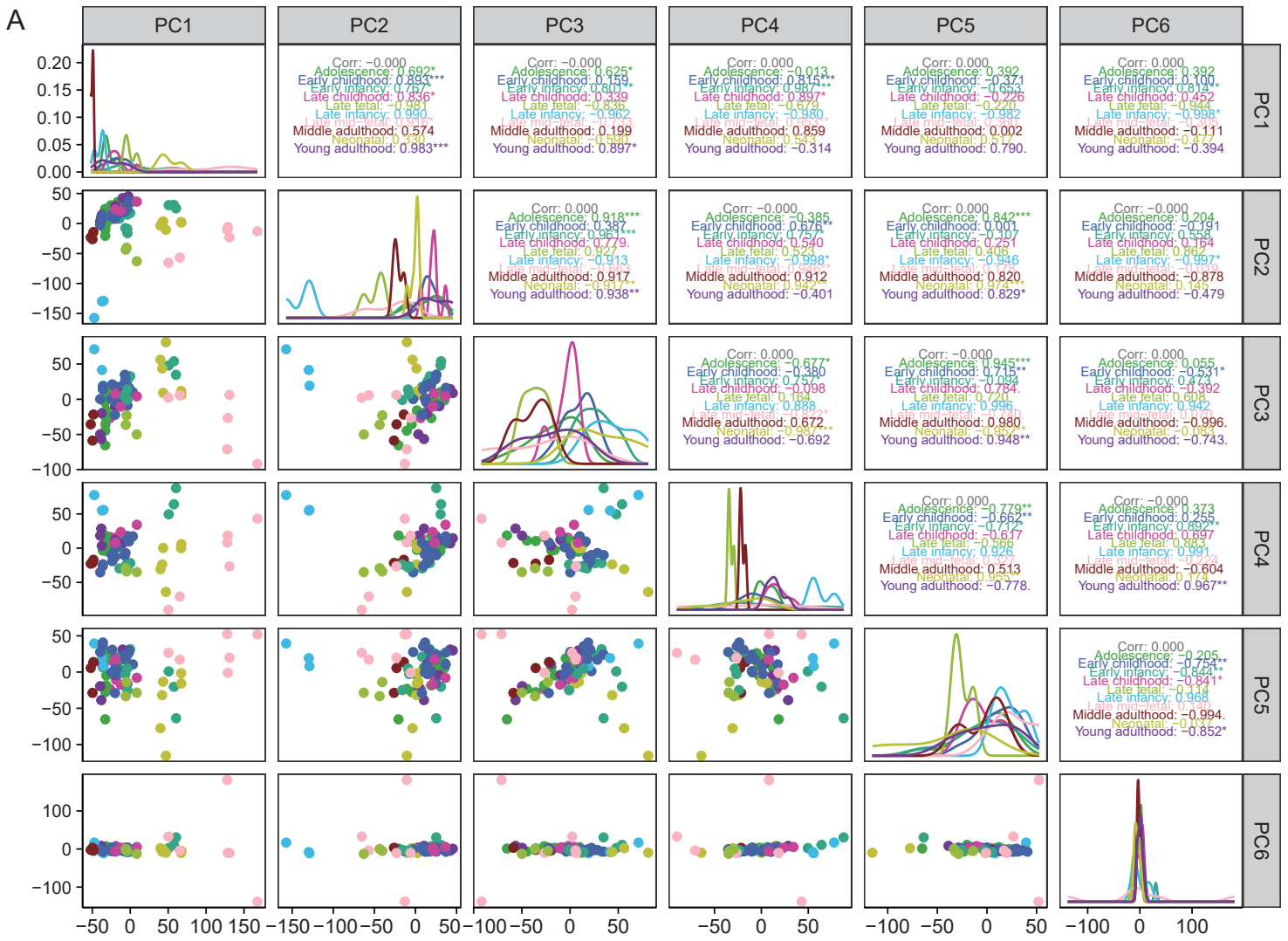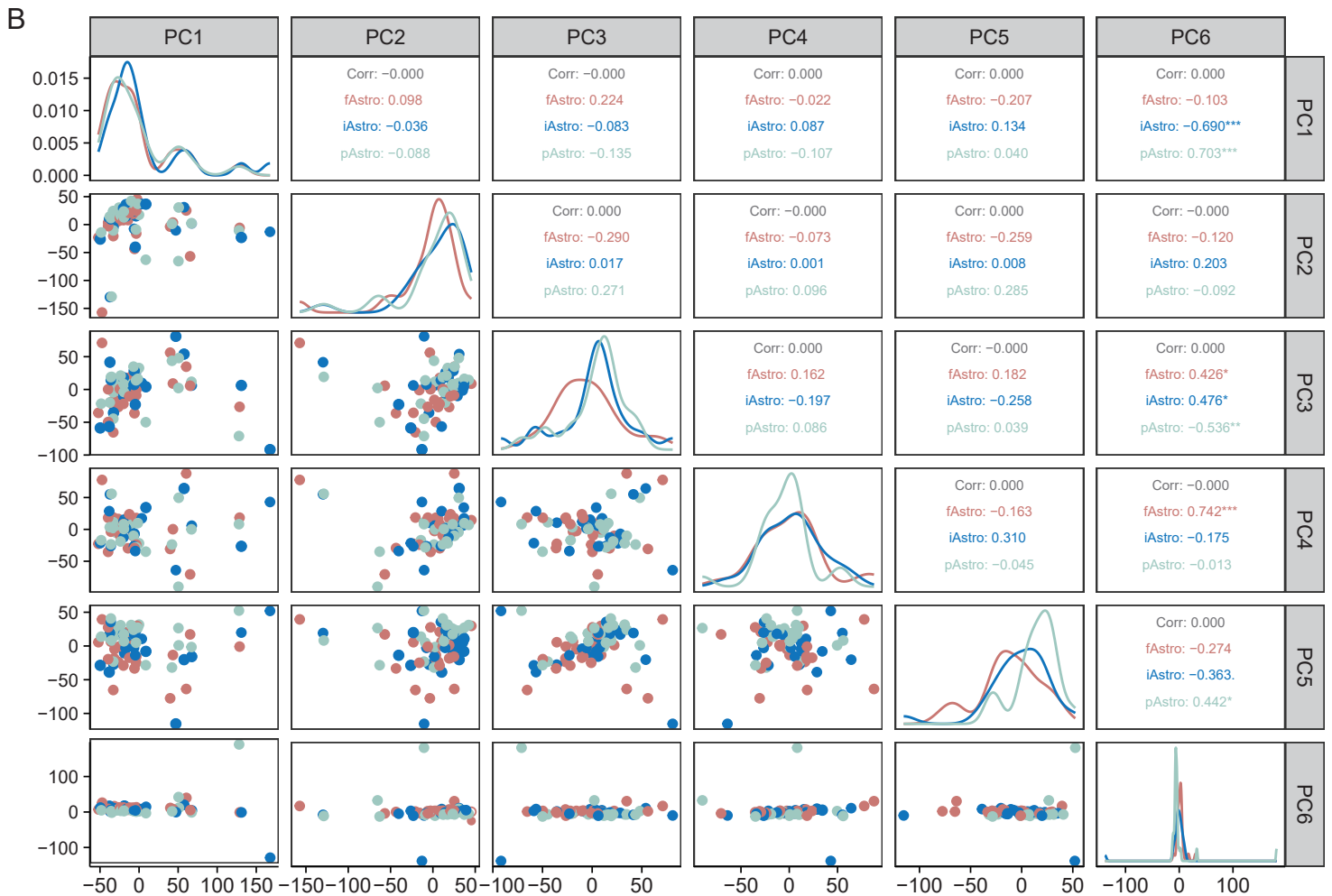

Supplement: Supplementary 1 — Supplementary Text Tables S1 to S5 Figs. S1 to S11 [file csbj.0083.f1.zip › FigS6.pdf]

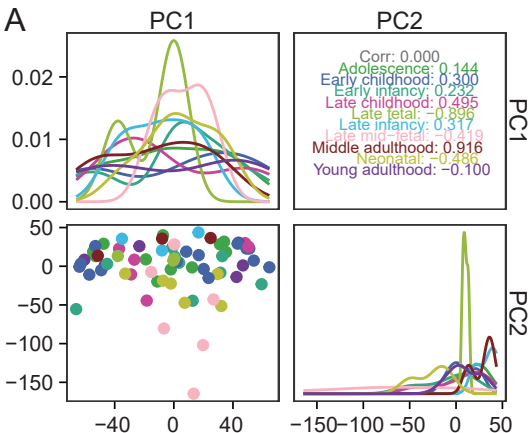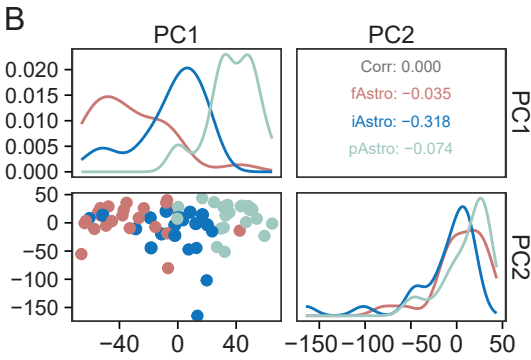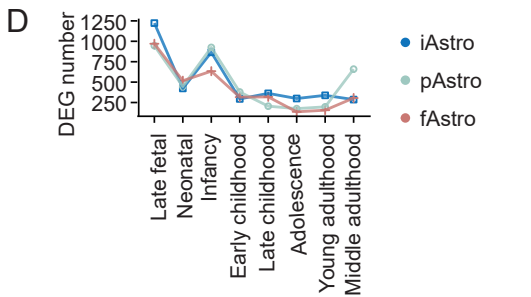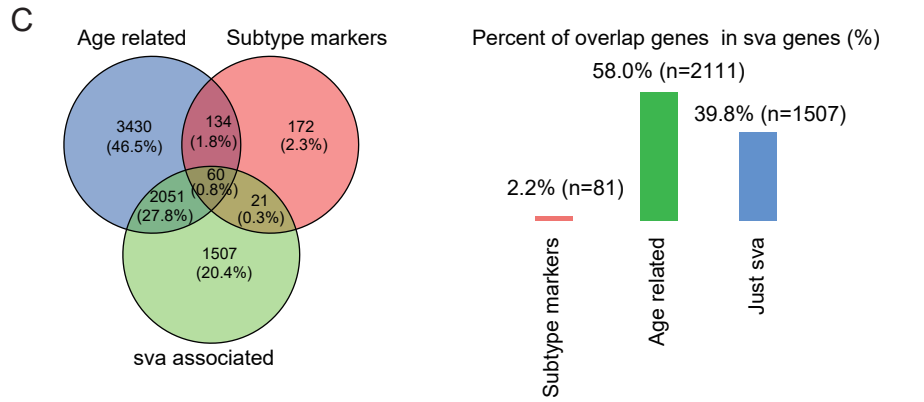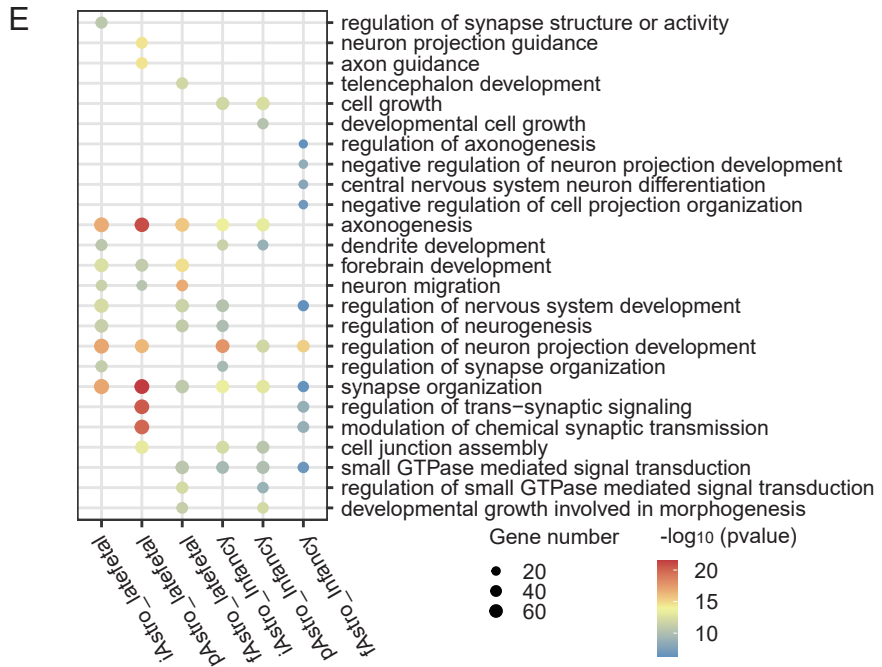

Supplement: Supplementary 1 — Supplementary Text Tables S1 to S5 Figs. S1 to S11 [file csbj.0083.f1.zip › FigS7.pdf]

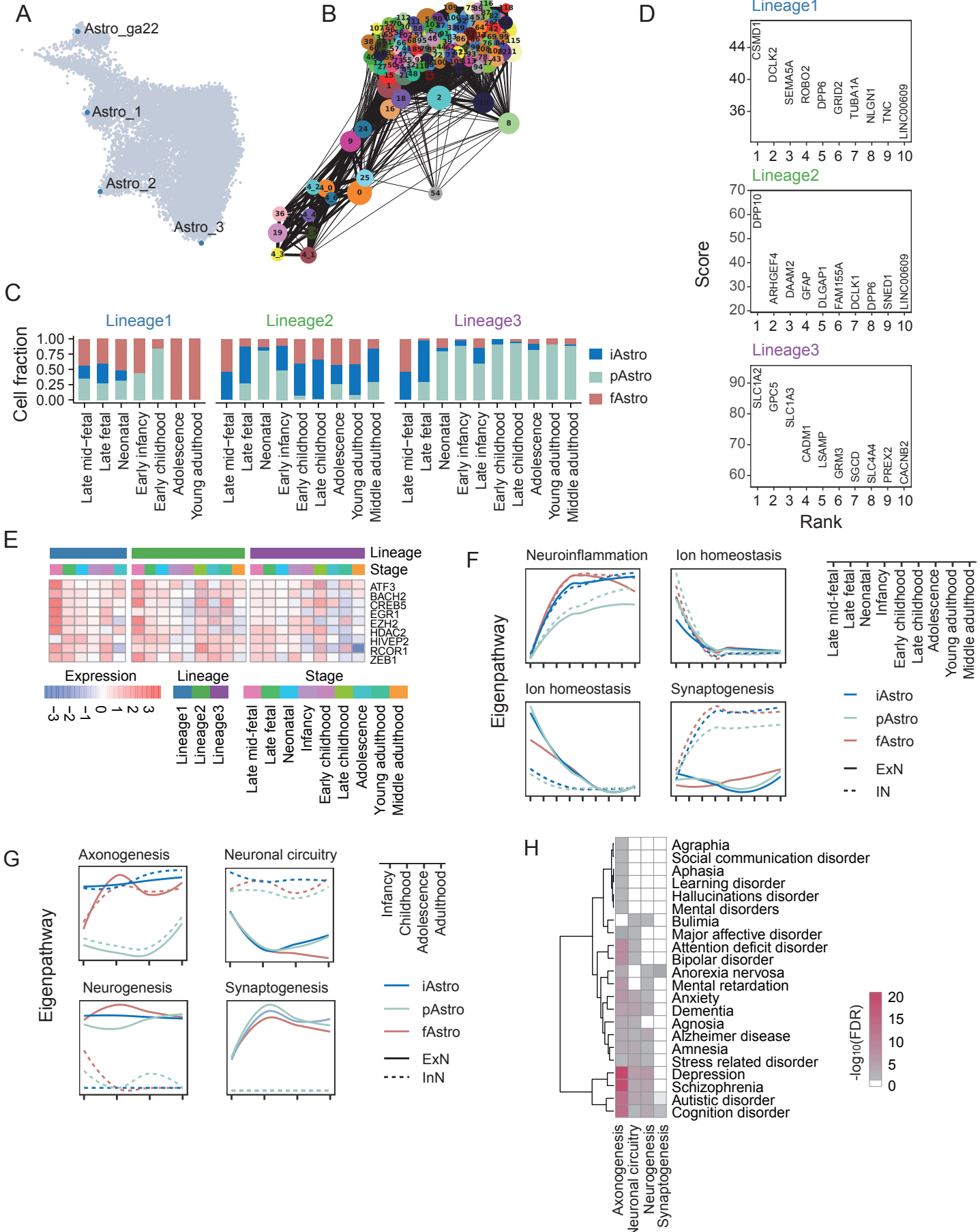

Supplement: Supplementary 1 — Supplementary Text Tables S1 to S5 Figs. S1 to S11 [file csbj.0083.f1.zip › FigS8.pdf]

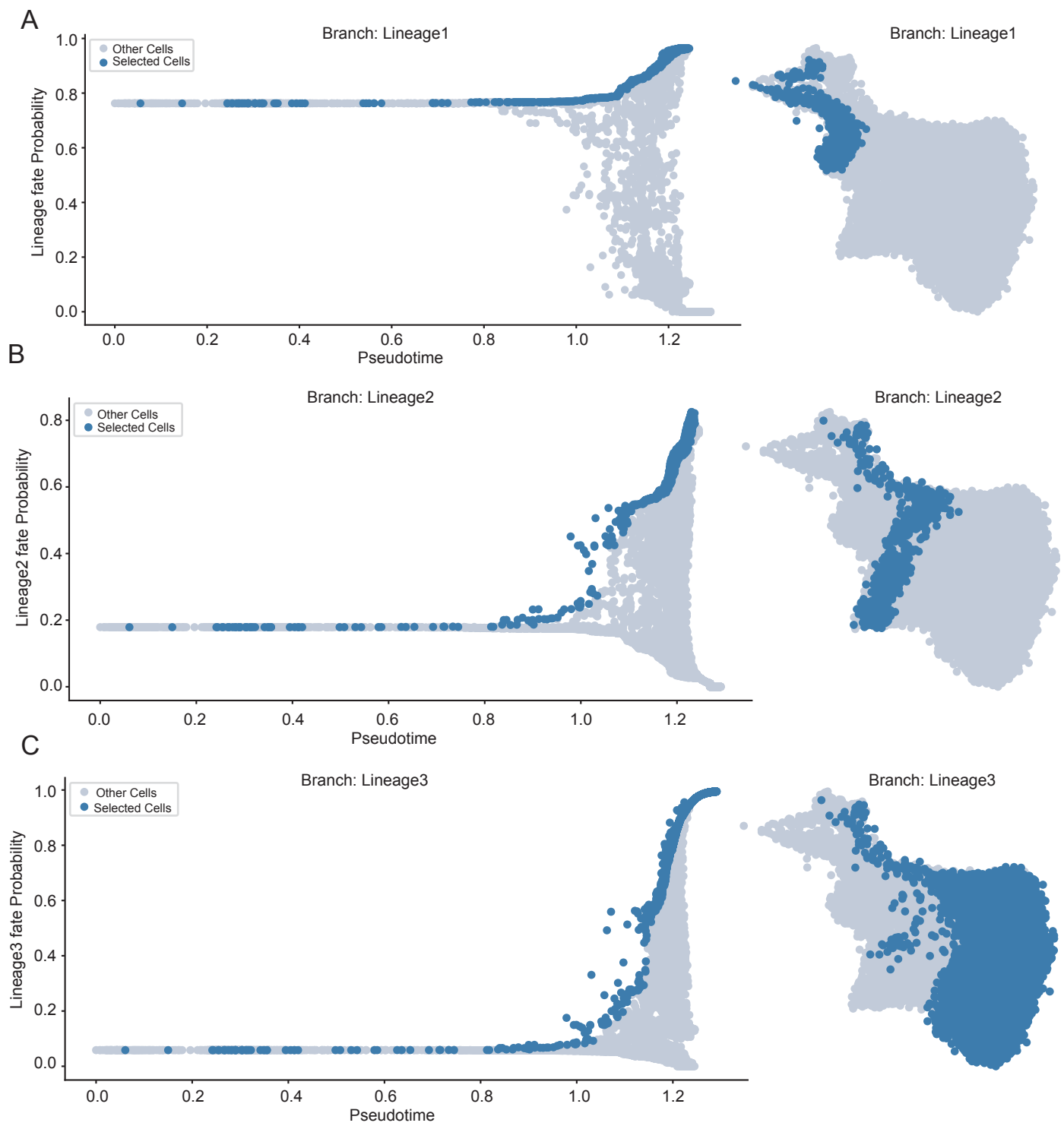

Supplement: Supplementary 1 — Supplementary Text Tables S1 to S5 Figs. S1 to S11 [file csbj.0083.f1.zip › FigS9.pdf]
